# The impact of chronic kidney disease on health-related quality of life (HRQoL): key insights from a hospital-based cross-sectional study

**DOI:** 10.1590/2175-8239-JBN-2024-0229en

**Published:** 2025-06-09

**Authors:** Gautam Sahu, Pooja Arora, Pramil Tiwari, Sanjay D’Cruz, Anita Tahlan

**Affiliations:** 1 National Institute of Pharmaceutical Education and Research (NIPER), Department of Pharmacy Practice, Mohali, Punjab, India.; 2 National Institute of Pharmaceutical Education and Research (NIPER), Department of Pharmacoinformatics, Mohali, Punjab, India.; 3 Government Medical College and Hospital (GMCH), Department of General Medicine, Chandigarh, India.; 4 Government Medical College and Hospital (GMCH), Department of Pathology, Chandigarh, India.

**Keywords:** Renal Insufficiency, Chronic, Quality of Life, Sociodemographic Factors, Comorbidity.

## Abstract

**Introduction:**

This study explores the health-related quality of life (HRQoL) in chronic kidney disease (CKD) patients, analyzing the impact of socio-demographic factors (such as age, education, and income) and clinical factors (including disease stage, comorbidities, and treatment type). The study aims to assess factors for improving patient outcomes and enhancing the overall well-being of CKD patients.

**Methods:**

This cross-sectional study, conducted at the nephrology clinic of a public teaching hospital, involved 560 randomly selected participants. The Kidney-Disease Quality of Life-Short Form (KDQOL-SF^TM^) questionnaire was used to collect data, analyzed via SPSS (version 20.0). Descriptive statistics were used to summarize baseline characteristics. Cronbach’s α was used to assess the reliability of questionnaire items. Differences in HRQoL scores between groups were examined using independent t-tests and ANOVA, while regression analysis was used to explore associations between variables.

**Results:**

The study analyzed the HRQoL in 560 CKD patients, who had a mean age of 53.32 years and a response rate of 81.27%. The mean HRQoL score was 32.03 ± 6.55. Males predominated in early CKD stages (72.5% in stages 1 and 2), while females were more prevalent in stage 4. Unemployment was high (73.6%). Patients > 50 years scored higher on the Burden of Kidney Disease (BKD) scores (26.73 ± 19.36), while younger patients showed better mental health outcomes. Freshly diagnosed patients had better scores across HRQoL domains than known cases. Education significantly predicted higher HRQoL (*p* < 0.001), but occupation and income did not. Hypertension (75.71%) and diabetes (39.64%) were common comorbidities, underscoring socio-economic influences on HRQoL in CKD.

**Conclusion:**

This study highlights the strong impact of socio-demographic and clinical factors, particularly education, employment, and disease stage, on CKD patients’ HRQoL. Early interventions and holistic CKD management, addressing clinical and socio-economic issues, are crucial for enhancing patient well-being.

## Introduction

In the contemporary landscape of healthcare, chronic kidney disease (CKD) presents significant challenges not only in terms of treatment but also in the overall well-being of affected patients^1,2^. This disorder, characterized by a progressive decline in kidney function, affects millions worldwide and severely impacts the physical, mental, and social dimensions of patients’ lives^3,4^. CKD patients experience a deterioration in their health-related quality of life (HRQoL), which stands out as a critical concern^5^. As healthcare shifts toward more patient-centered care, the importance of assessing and improving HRQoL in patients has gained increased attention from both clinical and research communities^6,7^.

On the clinical front, CKD stage, presence of comorbidities, and type of treatment, whether on dialysis or conservative management, also play a critical role in determining HRQoL^8,9^. Patients in advanced disease stages, particularly those undergoing dialysis, often report a significant decline in their physical and mental health. The physical demands of dialysis, coupled with the restrictions it imposes on daily life, create a profound sense of burden^10,11^. These patients may also face cognitive challenges, sleep disturbances, and feelings of dependency, all of which further erode HRQoL. Conversely, patients in earlier stages of the disease or those managed without dialysis are more likely to maintain a better HRQoL for longer, although they still face the looming prospect of disease progression^12,13^.

Assessing HRQoL in CKD patients is essential for providing a holistic understanding of how the disease affects patients beyond clinical parameters^14,15^. Furthermore, treatment modalities, particularly dialysis, introduce additional challenges such as time constraints, dietary restrictions, and emotional burdens, all of which negatively affect HRQoL^16,17^. In this context, understanding the factors that influence HRQoL in CKD patients becomes essential for tailoring interventions that can alleviate the burden of the disease and improve the overall HRQoL of the patients^18,19^.

Results available so far have indicated that several socio-demographic and clinical factors influence HRQoL among CKD patients. Age, gender, marital status, education level, employment status, income, and place of residence are some of the key variables that play a significant role in shaping outcomes. Additionally, disease-specific factors such as the stage of CKD, duration of illness, and type of treatment (dialysis or non-dialysis) further influence patients’ HRQoL scores. The multidimensional nature of HRQoL underscores the complexity of managing CKD, as it requires an integrated approach that goes beyond mere clinical management to include psychosocial support^20,21,22^.

Given the growing importance of HRQoL as a patient-centered outcome, several tools have been developed to measure it, one of the most widely used being the Kidney Disease Quality of Life-Short Form (KDQOL-SF^TM^) questionnaire. The use of such tools enables healthcare providers to gain a deeper understanding of how CKD impacts patients’ lives and to identify areas where targeted interventions can make a significant difference^23^.

This cross-sectional study aimed to assess the HRQoL among CKD patients and how various socio-demographic and clinical factors influence HRQoL. By systematically understanding the relationship between these factors and HRQoL, the study aims to assess factors for improving patient outcomes and enhancing the overall well-being of patients living with CKD.

## Methods

### Study Design and Patient Population

This cross-sectional study received approval from both the Institutional Ethics Committee (IEC/78/2023-RI) and the Hospital Ethics Committee (GMCH/IEC/2024/1137R).

The study was carried out at a Government Medical College and Hospital (GMCH) in Chandigarh, India, from April to October 2024, with a focus on CKD patients. From a pool of 689 patients who presented to the nephrology clinic during the study period, 560 were chosen based on predefined inclusion and exclusion criteria. Eligible participants were aged between 18 and 70 years and had provided written informed consent. Those with psychiatric disorders, hearing or speech impairments, a history of malignancy, recent or active blood loss, or participation in other drug trials, as well as pregnant and breastfeeding women, were excluded.

Detailed data were collected for each participant, which included personal information such as unit’s name, UHID number, demographic details (e.g., name, age, gender, address), as well as clinical and demographic factors like blood pressure, education, occupation, marital status, income, living conditions, social habits, health status, and other relevant variables.

### Data Collection Tool and Procedure

A structured and standardized self-administered questionnaire was employed, incorporating the Kidney Disease Quality of Life (KDQOL-SF™) survey. This tool consists of two parts: one addressing areas specific to end-stage renal disease (ESRD) and another comprising a 36-item general health survey (SF-36). While most participants completed the questionnaire independently, assistance was provided by researchers for those who required help. The ESRD section includes various scales such as 12 items on symptoms and problems (SP), 8 items on the effects of kidney disease (EKD), 4 items assessing the burden of kidney disease (BKD), 11 items covering physical component (PC), and 8 items focused on mental component (MC). The SF-36 component includes 10 items on physical functioning (PF), 4 on role limitations due to physical health (RP), 2 on bodily pain (BP), 5 on general health (GH), 5 on emotional well-being (EW), 3 on role emotional (RE), 2 on social functioning (SF), and 4 on energy and fatigue (EF). The responses are converted to a 0–100 scale, with higher scores indicating better health outcomes. Some items were pre-coded in such a manner that lower scores signify a more favorable health condition. In addition, demographic and clinical information was collected on factors such as gender, age, education, occupation, marital status, monthly income, residential status, and social habits^23^.

### Data Analysis

The data collected from the survey was analyzed using version 20.0 of the Statistical Package for the Social Sciences (SPSS) software. Descriptive statistics such as mean, standard deviations, percentages, and frequencies were computed to summarize sociodemographic and health-related characteristics. The distribution of participants’ responses of the KDQOL-SF™ was evaluated to identify the proportion of patients facing difficulties in each dimension. The descriptive data were presented in the form of percentages and frequencies. Internal consistency and reliability were assessed using Cronbach’s *α* coefficient, calculated for each subscale. Statistical tests, including independent *t*-tests for comparing the means of two groups and ANOVA for analyzing three or more groups, were employed to determine significant differences in demographics, health-related characteristics, and HRQoL. Additionally, regression analysis was conducted to verify associations between variables. A significance level of 5% (*p* < 0.05) was used to determine statistical significance.

## Results

**Figure 1:**
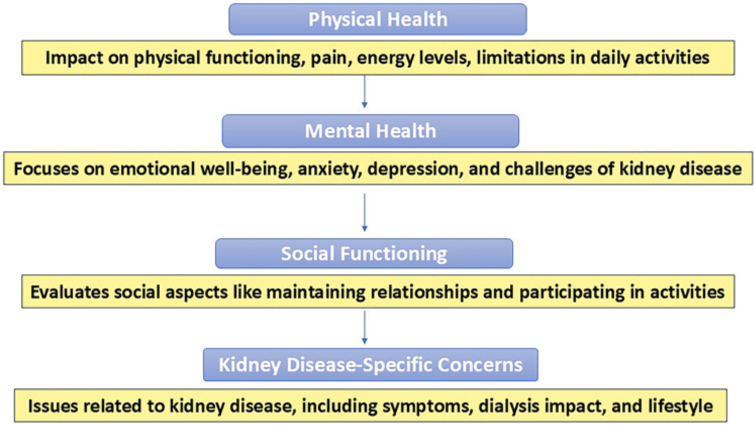
Four key domains of health in kidney disease.

The results of the study revealed significant variations in HRQoL among CKD patients based on socio-demographic and clinical characteristics. The overall impact of kidney disease on patients’ HRQoL can be categorized into four key domains: physical health, mental health, social functioning, and kidney disease-specific concerns (Figure 1). 

Table 1 provides a comprehensive overview of the socio-demographic characteristics of CKD patients across different stages. A considerable majority (56.6%) were treated on an outpatient department (OPD) basis, with stage 5 patients representing the highest proportion in this group. 59% of patients were over 50 years, particularly in stages 3 and 5. Male patients were more common in earlier stages (72.5% in stages 1 and 2) and the proportion of females was higher in stage 4 CKD.

**Table 1:** Comparative analysis of HRQoL scores across socio-demographic and disease-specific variables in patients with CKD

Variables	Stages of CKD				
	1 & 2 (n = 51)	3 (n = 70)	4 (n = 81)	5 (n = 358)	Total (n = 560)
Patient Type					
OPD	31 (60.8%)	37 (52.9%)	37 (45.7%)	212 (59.2%)	317 (56.6%)
IPD	20 (39.2%)	33 (47.1%)	44 (54.3%)	146 (40.8%)	243 (43.3%)
Age					
< 50	21 (41.2%)	24 (34.3%)	37 (45.7%)	146 (40.8%)	228 (40.7%)
> 50	30 (58.8%)	46 (65.7%)	44 (54.3%)	212 (59.2%)	332 (59.3%)
Gender					
Male	37 (72.5%)	39 (55.7%)	35 (43.2%)	207 (57.8%)	318 (56.8%)
Female	14 (27.5%)	31 (44.3%)	46 (56.8%)	151 (42.2%)	242 (43.2%)
Marital Status					
Married	46 (90.2%)	64 (91.4%)	74 (91.4%)	333 (93.0%)	517 (92.3%)
Unmarried	5 (9.8%)	6 (8.6%)	7 (8.6%)	25 (7.0%)	43 (7.7%)
Education					
Illiterate	18 (35.3%)	23 (32.9%)	30 (37.0%)	145 (40.5%)	216 (38.6%)
Matric Pass	27 (52.9%)	38 (54.3%)	39 (48.1%)	163 (45.5%)	267 (47.7%)
Graduate	6 (11.8%)	9 (12.9%)	12 (14.8%)	48 (13.4%)	75 (13.4%)
Beyond Graduate	0 (0.0%)	0 (0.0%)	0 (0.0%)	2 (0.6%)	2 (0.4%)
Occupation					
Student	4 (7.8%)	4 (5.7%)	4 (4.9%)	18 (5.0%)	30 (5.4%)
Employed	8 (15.7%)	14 (20.0%)	17 (21.0%)	79 (22.1%)	118 (21.1%)
Unemployed	39 (76.5%)	52 (74.3%)	60 (74.1%)	261 (72.9%)	412 (73.6%)
Monthly Income					
10,000	37 (72.5%)	56 (80.0%)	55 (67.9%)	266 (74.3%)	414 (73.9%)
25,000	9 (17.6%)	11 (15.7%)	24 (29.6%)	78 (21.8%)	122 (21.8%)
50,000	5 (9.8%)	2 (2.9%)	2 (2.5%)	9 (2.5%)	18 (3.2%)
1,00,000	0 (0.0%)	1 (1.4%)	0 (0.0%)	5 (1.4%)	6 (1.1%)
Residential Status					
Rural	19 (37.3%)	36 (51.4%)	39 (48.1%)	169 (47.2%)	263 (47.0%)
Urban	32 (62.7%)	34 (48.6%)	42 (51.9%)	189 (52.8%)	297 (53.0%)
Diagnosis					
Freshly Diagnosed	7 (13.7%)	7 (10.0%)	15 (18.5%)	45 (12.6%)	74 (13.2%)
Known Case	44 (86.3%)	62 (88.6%)	65 (80.2%)	303 (84.6%)	474 (84.6%)
Referred Case	0 (0.0%)	1 (1.4%)	1 (1.2%)	10 (2.8%)	12 (2.1%)
History of Past Disease					
Yes	43 (84.3%)	69 (98.6%)	71 (87.7%)	324 (90.5%)	507 (90.5%)
No	8 (15.7%)	1 (1.4%)	10 (12.3%)	34 (9.5%)	53 (9.5%)

The majority of patients across all stages were married (92.3%). Education levels showed that 47.7% of patients had completed secondary education, although illiteracy was more prevalent in the advanced stages, particularly stage 5 (40.5%). Unemployment was high across all stages, with 73.6% of patients being unemployed. Most of patients earned less than ₹10,000 per month (73.9%). Urban residents slightly outnumbered rural ones (53%), although rural patients were more prevalent in stages 3 and 4. Most patients were already known cases of CKD (84.6%), and a significant percentage (90.5%) had a history of diseases. These trends were particularly noticeable in the later stages of CKD.

### Prevalence of Social Habits in CKD Patients

The results show that the largest group of social habits consisted of alcohol users only, with 191 (34.10%) people. The second-largest group was smokers, with 166 (29.64%) people. Tobacco users made up the smallest group, with 108 (19.28%) people. The Others category, which included different habits, had 157 (28.03%) patients. This pattern suggests that alcohol consumption is the most common singular social habit among CKD patients, followed by smoking.

**Figure 2:**
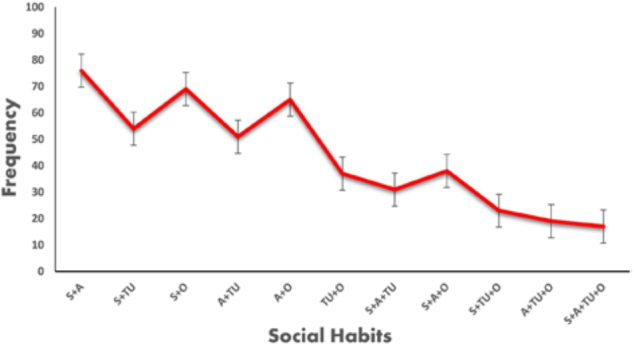
Distribution of social habits among CKD patients.

Figure 2 presents the prevalence of different combinations of social habits among CKD patients. The largest group consisted of patients who were both smokers and alcoholics (S+A) with 76 (13.57%) patients, followed by those who were smokers and fell into the ‘Others’ category (S+O) with 69 (12.32%) Patients. Conversely, the group with the least representation was the one with all four habits—smoking, alcohol consumption, tobacco use, and other habits (S+A+TU+O), which included only 17 patients (3.03%). The variability in the number of patients for each group suggests that while smoking and alcohol consumption are the most common habits among CKD patients, the combination of all four behaviors is less prevalent. These patterns may have implications for patient education and intervention programs aimed at reducing lifestyle-related risks associated with CKD.

Abbreviations – Smoker (S), Tobacco User (TU), Alcoholic (A), Others (O). Notes – *Other includes – Dietary habits, physical inactivity, inadequate hydration, drug users, and nonadherence to medication.

### Leading CKD Comorbidities

**Figure 3:**
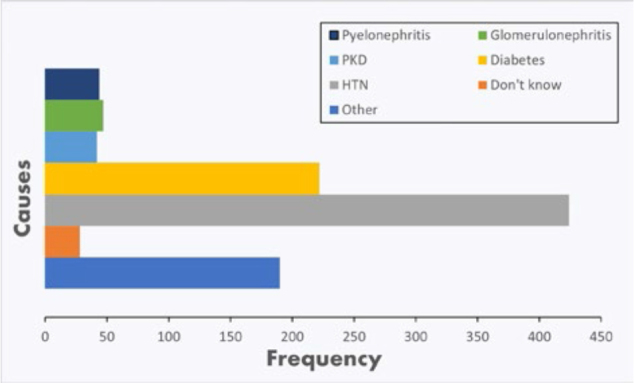
Causes of kidney disease.

The data from Figure 3 shows that the most common condition among CKD patients was hypertension (HTN), affecting 424 (75.71%) patients. Diabetes was the second most common condition, with 222 (39.64%) patients. 42 (7.50%) patients had other kidney-related conditions, such as polycystic kidney disease (PKD), while 47 (8.39%) had glomerulonephritis, and 44 had pyelonephritis (7.85%). Additionally, 190 (33.92%) patients were classified under “Other” conditions, and 28 (5.00%) patients indicated that they “Didn’t know” their underlying condition. This distribution suggests that hypertension and diabetes are the predominant comorbidities in CKD patients, highlighting the need for focused management and intervention strategies targeting these conditions.

### Disease-Specific Characteristics: ESRD Focused Area

The analysis of HRQoL scores among CKD patients revealed significant differences based on socio-demographic and disease-specific characteristics. OPD patients demonstrated slightly better symptom management (70.03 ± 13.43) and mental health (63.10 ± 9.45) compared to IPD (in-patients), who scored marginally higher in the physical component (53.57 ± 7.36). However, OPD patients reported a significantly higher BKD (31.55 ± 21.13) than IPD patients (26.52 ± 19.42) (*p* = 0.004). Patients aged > 50 years showed a greater BKD (26.73 ± 9.36) compared to younger patients, who had better scores in the MC. Gender differences were observed only in the PC, where males scored higher than females (*p* = 0.004). Unmarried patients exhibited a significantly higher disease burden (40.31 ± 27.78) but reported better mental health compared to married patients (*p <* 0.001). Graduates reported better physical and mental health outcomes (*p <* 0.001). Employed patients scored higher in mental health but also experienced a greater BKD compared to unemployed patients (*p <* 0.001). Income was also a significant factor, with higher-income patients reporting a reduced BKD. Urban patients experienced a higher BKD than rural patients, although the differences in physical and mental health scores were not significant. Freshly diagnosed patients reported the highest BKD (*p <* 0.001), while those without a history of past disease showed better MC. These findings underscore the importance of age, marital status, education, employment, income, and disease status in shaping the HRQoL for CKD patients, suggesting that tailored interventions should be used to address these specific vulnerabilities (Table S1).

### ESRD Domains and Scores Across CKD Stages

A detailed analysis of the mean scores and reliability was performed for various domains associated with ESRD across different stages of CKD. The SP list, which had a relatively low reliability score (Cronbach’s *α* = 0.365), showed a significant variation across CKD stages, with stage 3 patients reporting the highest mean score (75.20 ± 11.37) and stage 1 and 2 patients, the lowest (69.32 ± 12.78), indicating a statistically significant difference (*p* = 0.023). In contrast, the EKD domain, with a high reliability score (Cronbach’s *α* = 0.847), did not show significant differences across stages, with scores ranging from 59.34 ± 10.81 in stage 5 to 62.63 ± 9.50 in stage 3 (*p* = 0.078). The BKD domain had moderate reliability (Cronbach’s *α* = 0.550), and no significant differences were observed between stages (*p* = 0.550). Similarly, the PC had low reliability (Cronbach’s *α* = 0.025), with no significant differences across stages (*p* = 0.215). The MC, with moderate reliability (Cronbach’s *α* = 0.661), also did not show significant differences across stages (*p* = 0.133). These results highlight that while symptoms vary significantly across CKD stages, other aspects such as the effects and burden of the disease and the physical and mental components remain relatively stable throughout disease progression (Table S2).

### Comparison of 36-Item HRQoL Scores Across CKD Stages

Table 2 presents the analysis of various HRQoL domains in CKD patients across different stages, using a 36-item scale. The PF domain, with a high reliability score (Cronbach’s *α* = 0.883), did not show significant differences between stages (*p* = 0.370), with stage 3 patients reporting the highest scores. Similarly, the RP domain, which measures limitations in daily activities, had high reliability (Cronbach’s *α* = 0.782) and no significant stage-wise differences (*p* = 0.628). BP, with high reliability (Cronbach’s *α* = 0.851), also remained stable across stages (*p* = 0.889), though patients in stages 1 and 2 reported slightly higher pain levels. GH was consistent across all stages (*p* = 0.919; Cronbach’s *α* = 0.576). EW had lower reliability (Cronbach’s *α* = 0.266) but did not show significant differences (*p* = 0.254), although stage 1 and 2 patients reported higher scores. The RE domain, which had a high reliability score (Cronbach’s *α* = 0.782), showed significant variability across stages (*p* = 0.034), indicating that emotional functioning is more impacted by CKD progression. SF and EF did not show significant differences between stages, with moderate and low-reliability scores, respectively. These findings suggest that while most HRQoL measures remain stable across CKD stages, emotional functioning is more affected as the disease progresses.

**Table 2:** 36-Item domains and mean summary scores of patients in different stages of CKD patients

Scales	No. of Items	Cronbach’s *α*	Stages of CKD					
			1 & 2 (n = 51)	3 (n = 70)	4 (n = 81)	5 (n = 358)	Total (n = 560)	*p*-value
PF	10	0.883	39.12 ± 20.53	45.00 ± 14.81	43.52 ± 21.14	42.63 ± 18.24	42.73 ± 18.53	0.370
RP	4	0.782	22.06 ± 34.14	15.36 ± 26.33	18.83 ± 29.45	17.25 ± 29.60	17.68 ± 29.60	0.628
BP	2	0.851	24.18 ± 37.16	19.52 ± 33.32	23.04 ± 34.01	22.06 ± 34.66	22.08 ± 34.56	0.889
GH	5	0.576	50.02 ± 16.11	50.67 ± 10.43	51.06 ± 15.88	49.89 ± 15.24	50.17 ± 14.88	0.919
EW	5	0.266	67.61 ± 21.47	63.56 ± 17.52	62.06 ± 21.80	66.16 ± 19.45	65.37 ± 19.73	0.254
RE	3	0.782	55.14 ± 8.47	55.26 ± 6.57	52.40 ± 9.43	55.68 ± 9.42	55.10 ± 9.08	0.034
SF	2	0.438	40.69 ± 8.18	43.14 ± 7.57	42.84 ± 8.94	41.73 ± 8.68	41.97 ± 8.55	0.311
EF	4	−0.238*	33.24 ± 18.10	38.64 ± 12.73	37.16 ± 16.16	36.87 ± 16.84	36.80 ± 16.41	0.344

Abbreviations – PF: Physical Functioning; RP: Role Physical; BP: Bodily Pain; GH: General Health; EW: Emotional Well-Being; RE: Role Emotional; SF: Social Functioning; EF: Energy/Fatigue.

Notes – *The value is negative due to a negative average covariance among items. This violates reliability model assumptions.

### Impact of Patient Characteristics on HRQoL Scores

A comparative analysis of HRQoL scores across various domains in patients with CKD was performed based on socio-demographic and disease-specific variables. The OPD patients scored significantly higher in physical functioning (*p* = 0.008) and bodily pain (*p* = 0.002) compared to IPD patients. EW (*p <* 0.001) was also higher in OPD patients, while other domains showed no significant difference. Patients aged < 50 years had significantly higher scores across multiple domains, including PF (*p <* 0.001), RP (*p <* 0.001), GH (*p* = 0.003), and EW (*p <* 0.001), among others, compared to patients > 50 years old. While there were no major differences between males and females in most domains, RP (*p* = 0.017) and RE (*p* = 0.020) were significantly higher in males. Unmarried patients scored significantly higher in PF (*p <* 0.001), RP (*p <* 0.001), GH (*p* = 0.040), and EW (*p* = 0.003) compared to married patients. Higher educational attainment was associated with significantly better scores in almost all domains, including PF (*p <* 0.001), RP (*p <* 0.001), and GH (*p <* 0.001). Employed and student patients reported significantly better scores in most domains compared to unemployed patients, with PF (*p <* 0.001), RP (*p <* 0.001), and BP (*p <* 0.001) showing the largest differences. Higher income was linked to significantly better outcomes in PF (*p* = 0.003), RP (*p <* 0.001), and GH (*p <* 0.001). Urban patients scored higher in PF (*p <* 0.001), BP (*p <* 0.001), and EW (*p* = 0.026), while rural patients had lower scores across these domains. Freshly diagnosed patients reported significantly higher scores in all domains compared to known cases, with PF (*p <* 0.001) and RP (*p <* 0.001) showing the largest differences. Patients without a history of past disease had significantly better scores in PF (*p <* 0.001), RP (*p <* 0.001), and EW (*p* = 0.005). In conclusion, the analysis reveals that socio-demographic factors like age, marital status, education, and income, along with disease-specific characteristics, significantly influence the HRQoL in CKD patients. Younger, unmarried, educated, and employed patients, as well as those without a history of past disease, tend to report better health outcomes across multiple domains (Table S3).

### Risk Behaviors by Demographics

Table 3 presents a comparative analysis of risk behavior patterns, such as smoking, alcohol consumption, tobacco use, and other unhealthy behaviors, among CKD patients, categorized by age and gender. Among males, those aged > 50 years exhibited a higher prevalence of smoking (39.2%) compared to younger males aged < 50 years (31.1%). However, alcohol consumption was more common in younger males (52.3%) than in older males (37.6%). Tobacco use, in contrast, was significantly higher in males aged > 50 years (24.7%) compared to their younger counterparts (6.1%). Other unhealthy behaviors, such as poor dietary habits, physical inactivity, and non-adherence to medication, were slightly more prevalent in older males (30.1%) than in younger ones (28.8%). Among females, smoking and alcohol consumption were considerably lower than in males. Younger females (< 50 years) had a lower prevalence of smoking (9.4%) and alcohol use (15.6%) than their male counterparts. Similarly, older females (> 50 years) had low rates of smoking (9.6%) and alcohol use (10.3%), with only slight differences compared to younger females. Tobacco use was marginally higher in older females (8.2%) compared to younger females (7.3%). Other unhealthy behaviors were more common in older females (26.0%) than in younger females (21.9%). In conclusion, males showed a higher prevalence of smoking, alcohol, and tobacco use than females across all age groups. Additionally, risk behaviors tend to increase with age in both genders. These findings highlight the importance of targeted interventions to reduce risk behaviors, particularly among older male CKD patients, who exhibited higher rates of tobacco and alcohol use.

**Table 3:** Risk behavior patterns by age and gender

Variables	Age (Years)	Smokers N (%)	Alcoholic N (%)	Tobacco user N (%)	Others N (%)
Gender					
Male	< 50	41 (31.1)	69 (52.3)	8 (6.1)	38 (28.8)
	> 50	73 (39.2)	70 (37.6)	46 (24.7)	56 (30.1)
*p*-value		0.134	0.010	< 0.001	0.799
Female	< 50	9 (9.4)	15 (15.6)	7 (7.3)	21 (21.9)
	> 50	14 (9.6)	15 (10.3)	12 (8.2)	38 (26.0)
*p*-value		0.956	0.217	0.793	0.462

Note – Other includes: poor dietary habits, physical inactivity, inadequate hydration, drug user, and non-adherence to medication.

### Comorbidity Patterns by Age and Gender

Table 4 provides a detailed analysis of the prevalence of comorbidities such as HTN, diabetes, polycystic kidney disease (PKD), glomerulonephritis, pyelonephritis, and other related conditions, categorized by age and gender among CKD patients. Among males, hypertension was more common in those aged > 50 years (79.6%) compared to those aged < 50 years (72.0%). Diabetes showed a statistically significant increase in prevalence among older males (45.7%) compared to younger ones (32.6%) (*p* = 0.019). PKD and glomerulonephritis were slightly more frequent in younger males, although these differences were not statistically significant. Pyelonephritis was significantly more prevalent in younger males (15.2%) compared to those > 50 years (5.9%) (*p* = 0.006). For females, the prevalence of hypertension was higher in those > 50 years (80.1%) compared to younger females (66.7%) (*p* = 0.018). Diabetes was significantly more prevalent in older females (47.3%) than in those < 50 (26.0%) (*p <* 0.001) years. PKD and glomerulonephritis were more common in younger females, with PKD showing a significantly higher rate among younger females (14.6%) compared to older females (4.1%) (*p* = 0.004). Pyelonephritis was slightly more prevalent among younger females (8.3%) than older ones (3.4%), but the difference was not statistically significant. In conclusion, the analysis revealed that hypertension and diabetes were more prevalent among older CKD patients, particularly in females. Younger males and females tend to have a higher prevalence of pyelonephritis and PKD. These findings suggest that the distribution of comorbidities varies with age and gender simultaneously, highlighting the need for tailored medical interventions to address the specific comorbid risks in different demographic groups.

**Table 4:** Comparative analysis of comorbidities

Variables	Age (Years)	HTN N (%)	Diabetes N (%)	PKD N (%)	Glomerulonephritis N (%)	Pyelonephritis N (%)	Others N (%)	Don’t Know N (%)
Gender								
Male	< 50	95 (72.0)	43 (32.6)	12 (9.1)	15 (11.4)	20 (15.2)	40 (30.3)	7 (5.3)
	> 50	148 (79.6)	85 (45.7)	10 (5.4)	12 (6.5)	11 (5.9)	56 (30.1)	9 (4.8)
*p*-value		0.116	0.019	0.198	0.112	0.006	0.970	0.852
Female	< 50	64 (66.7)	25 (26.0)	14 (14.6)	8 (8.3)	8 (8.3)	39 (40.6)	2 (2.1)
	> 50	117 (80.1)	69 (47.3)	6 (4.1)	12 (8.2)	5 (3.4)	55 (37.7)	10 (6.8)
*p*-value		0.018	< 0.001	0.004	0.975	0.098	0.645	0.095

Note – Other includes: UTI, pancreatitis, drug use, hepatitis, pedal edema, and obstruction.

### Relevance of Factors Influencing HRQoL

The regression analysis in Table 5 shows the association between HRQoL and various factors. The model explains about 14.1% of the variation in HRQoL, with the overall model being statistically significant (*F* = 8.147, *p* < 0.001). Among the variables, patient type shows a significant negative impact on HRQoL (*B* = −8.79, *p* = 0.002), suggesting that patients in different categories (likely inpatients) have lower HRQoL scores compared to others. Age, although having a positive coefficient (*B* = 2.82), did not significantly influence HRQoL (*p* = 0.394), nor did gender (*B* = −0.96, *p* = 0.738). Similarly, marital status, while having a positive effect (*B* = 3.91), was not statistically significant (*p* = 0.613), indicating it had little impact on HRQoL. Education, however, emerges as a significant positive predictor (*B* = 10.71, *p* < 0.001), demonstrating that higher education levels are strongly associated with better HRQoL. In contrast, occupation and monthly income, although positively associated with HRQoL (*B* = 1.62 and *B* = 2.97, respectively), were not statistically signi­ficant (*p* = 0.683 and *p* = 0.349), suggesting that employment status and income levels do not significantly affect HRQoL in this sample. Residential status also showed no significant effect (*B* = 3.66, *p* = 0.244). The type of diagnosis played a crucial role in determining HRQoL, with a significant negative impact (*B* = −13.36, *p* = 0.007), indicating that certain diagnoses are associated with poorer HRQoL. Interestingly, the history of past disease positively influenced HRQoL (*B* = 13.49, *p* = 0.04), possibly because patients with a history of illness may have better coping mechanisms or management strategies. The stage of the disease, however, did not show a significant effect on HRQoL (*B* = 0.020, *p* = 0.987), suggesting that disease progression alone does not substantially alter HRQoL in this model. Overall, education and diagnosis emerged as the most significant factors affecting HRQoL, while other variables such as age, gender, occupation, and income show minimal influence.

**Table 5:** Regression analysis showing the association between hrqol and various demographic and clinical variables

Variables	*B*	Std. Error	*t*	Sig.
Patient Type	−8.79	2.84	−3.09	0.002
Age	2.82	3.31	0.85	0.394
Gender	−0.96	2.89	−0.33	0.738
Marital Status	3.91	7.73	0.50	0.613
Education	10.71	2.58	4.13	< 0.001
Occupation	1.62	3.96	0.40	0.683
Monthly Income	2.97	3.17	0.93	0.349
Residential Status	3.66	3.14	1.16	0.244
Diagnosis	−13.36	4.90	−2.72	0.007
History of Past Disease	13.49	6.55	2.05	0.040
Stages	0.020	1.23	0.017	0.987

Notes – *R* ^2^ = 0.141, *F* = 8.147, and *p* < 0.001.

## Discussion

The results of this study reveal important insights into the HRQoL of patients with CKD, highlighting the profound impact of socio-demographic and clinical factors on patient outcomes. The findings underscore the multifaceted nature of CKD management, wherein not only clinical indicators but also social and psychological dimensions must be considered to comprehensively improve patient well-being.

A significant finding of this study was the variation in HRQoL scores across different CKD stages, with patients in advanced stages, particularly those on dialysis, experiencing notably lower HRQoL compared to those in earlier stages^4,24^. This decline, especially in physical and emotional well-being, is largely attributed to the cumulative impact of CKD-related symptoms such as fatigue, pain, and cognitive impairments^25^. Additionally, the demanding nature of dialysis, including frequent treatments, dietary restrictions, and reduced physical activity, further exacerbates the deterioration in quality of life. In this study, inpatients exhibited lower scores in the BKD and PC domains, which aligns with previous research linking advanced CKD stages to significantly diminished HRQoL^25,26,27,28^.

Moreover, socio-demographic factors played a pivotal role in shaping HRQoL outcomes. Age emerged as a significant determinant, with younger patients (< 50 years) reporting better physical functioning and emotional well-being compared to older patients (> 50 years)^19,29^. This could be due to the greater physical resilience and psychological adaptability of younger patients, whereas older patients may face more comorbid conditions and physical limitations that impair their HRQoL^30,31^. Interestingly, unmarried patients exhibited higher scores in several domains, including emotional well-being and physical functioning, compared to their married counterparts. This may be attributed to differences in social support systems or the added responsibilities and stress that come with family life, particularly for those managing a chronic illness^32^.

Educational attainment and employment status also significantly influenced HRQoL scores. Patients with higher educational levels and those who were employed reported better overall HRQoL^33,34^. These groups likely benefit from better access to healthcare resources, greater health literacy, and a sense of purpose or routine through employment^35^. In contrast, unemployed patients and those with lower education levels were more likely to experience financial constraints, which can limit access to care and increase psychological stress, further diminishing their HRQoL^36^. Additionally, income affected HRQoL scores, with higher-income patients demonstrating better outcomes in domains such as physical fun­ctioning and bodily pain. This finding highlights the socioeconomic inequalities that persist in healthcare access and outcomes among CKD patients^35,37^.

The influence of clinical factors such as disease duration and presence of comorbidities such as hypertension and diabetes were strongly associated with lower HRQoL, especially in older patients^19,38^. The cumulative burden of managing multiple chronic conditions contributes to worse physical and general health outcomes. Freshly diagnosed patients, however, reported better HRQoL, likely due to fewer symptoms and psychological strain in the early stages of CKD^24^. This highlights the need for early intervention and continuous support to maintain HRQoL as the disease advances^39^.

In summary, the study demonstrates that CKD management requires a personalized approach that considers disease stage, socio-demographic factors, and comorbidities. Tailored interventions, such as enhanced psychosocial support for younger patients and integrated care for older patients with multiple conditions, are essential. Addressing socioeconomic barriers is also crucial to reducing disparities in HRQoL. Ultimately, improving HRQoL in CKD patients requires a holistic, patient-centered approach that goes beyond clinical care to address broader psychosocial and economic challenges.

### Limitations

The single-center nature of this study may limit the generalizability of findings. The lack of longitudinal follow-up restricts insight into changes in HRQoL over time, and the absence of data on the impact of emotional and psychological support may affect the validity of the results.

### Strengths

A major strength of this study lies in its adequate sample size, which enhances the reliability and robustness of the findings. The study’s focus on socio-demographic and clinical variables provides a comprehensive understanding of how various factors influence HRQoL, offering insights into targeted interventions. Moreover, the cross-sectional design highlights varying needs across CKD stages.

### Future Research Directions

Future work should consider a longitudinal approach for tracking HRQoL over time, multi-center designs for broader applicability, and examining the role of mental health interventions and social support in enhancing HRQoL. Integrating qualitative insights would further refine patient-centered interventions.

## Conclusions

This study underscores the significant impact of socio-demographic and clinical factors on HRQoL in patients with CKD by analyzing key determinants such as age, education, employment status, income level, and comorbid conditions. This study provides a deeper understanding of the broader challenges faced by CKD patients and identify potential areas for targeted interventions.

The findings highlight the substantial decline in HRQoL as the disease progresses, particularly in advanced stages requiring dialysis. Patients in later stages experience notable deterioration in physical, emotional, and social well-being, emphasizing the necessity of a comprehensive approach to CKD management.

Socio-demographic factors play a critical role in shaping HRQoL outcomes, with younger, educated, and employed patients reporting better HRQoL, whereas older, unemployed, and lower-income patients face greater difficulties.

Furthermore, the presence of comorbid conditions such as hypertension and diabetes further exacerbates the decline in HRQoL, particularly among older patients. Additionally, freshly diagnosed patients exhibited better HRQoL compared to those with a longer disease history, emphasizing the importance of early detection and timely intervention.

This study concludes that CKD management must extend beyond clinical interventions to encompass psychosocial, economic, and emotional factors. A comprehensive, patient-centered approach that inte­grates educational initiatives, psychosocial support, and improved healthcare access is essential for enhancing HRQoL. Ultimately, a multidimensional framework combining medical, psychological, and social care is crucial to addressing the complex needs of CKD patients and improving their overall well-being.

## Data Availability

This study involved the analysis of publicly accessible datasets. The datasets utilized and/or examined in the current research can be obtained from the corresponding author upon a reasonable request.

## Supplementary Material

The following online material is available for this article:

Table S1 – Analysis of HRQoL Scores Based on Disease-Specific Characteristics and Comparative Statistical Evaluation of Mean Scores in ESRD-Focused Areas Among CKD Patients, Stratified by Socio-Demographic Variables.

Table S2 – ESRD Targeted Domains and Mean Summary Scores of Patients in Different Stages of CKD.

Table S3 – HRQoL Scores by Disease-Specific Characteristics, and Comparative Statistical Analysis of Mean Scores of SF-36 Items among Patients with CKD according to Socio-Demographic Variables.
