# Supplementary material for: The impact of chronic kidney disease on health-related quality of life (HRQoL): key insights from a hospital-based cross-sectional study
Source: J Bras Nefrol. 2025 Jun 9;47(3):e20240229. doi: 10.1590/2175-8239-JBN-2024-0229en (PMC12176062; doi:10.1590/2175-8239-JBN-2024-0229en)
Supplement: Supplementary file 1 [file 2175-8239-jbn-47-3-e20240229-suppl1.pdf]

Braz. J. Nephrol.
https://doi.org/10.1590/2175-8239-JBN-2024-0229en

Supplementary Material to "The impact of chronic kidney disease on health-related quality of life (HRQoL): key insights from a hospital-based cross-sectional study"

Table S1 - Analysis of HRQoL Scores Based on Disease-Specific Characteristics and Comparative Statistical Evaluation of Mean Scores in ESRD-Focused Areas Among CKD Patients, Stratified by Socio-Demographic Variables.

Variables	N (%)	SP	EKD	BKD	PC	MC
**Patient Type**						
OPD	317	70.03 ± 13.43	60.72 ± 11.89	31.55 ± 21.13	54.40 ± 7.07	63.10 ± 9.45
IPD	243	71.56 ± 13.11	59.34 ± 8.78	26.52 ± 19.42	53.57 ± 7.36	61.74 ± 9.75
p-value	..	0.178	0.131	0.004	0.175	0.097
**Age**						
< 50	228	71.07 ± 13.19	58.42 ± 12.12	33.21 ± 21.62	54.56 ± 7.58	64.83 ± 9.16
> 50	332	70.43 ± 13.39	61.29 ± 9.38	26.73 ± 19.36	53.69 ± 6.92	60.92 ± 9.58
p-value	..	0.581	0.002	< 0.001	0.162	< 0.001
**Gender**						
Male	318	70.93 ± 13.33	60.24 ± 10.48	30.30 ± 21.51	54.80 ± 7.70	63.06 ± 10.39
Female	242	70.37 ± 13.28	59.96 ± 10.92	28.15 ± 19.16	53.05 ± 6.38	61.80 ± 8.41
p-value	..	0.621	0.758	0.220	0.004	0.124
**Marital Status**						
Married	517	70.65 ± 13.20	60.42 ± 10.18	28.46 ± 19.58	53.88 ± 7.27	61.99 ± 9.25
Unmarried	43	71.22 ± 14.64	56.54 ± 15.04	40.31 ± 27.78	56.05 ± 6.07	68.84 ± 11.45
p-value	..	0.788	0.022	< 0.001	0.058	< 0.001
**Education**						
Illiterate	216	67.31 ± 13.22	59.80 ± 10.78	27.14 ± 18.02	52.10 ± 6.02	58.94 ± 9.20
Matric Pass	267	71.80 ± 12.68	60.26 ± 10.05	31.76 ± 21.37	53.91 ± 6.65	64.27 ± 9.11
Graduate	75	76.77 ± 13.06	61.16 ± 11.96	26.55 ± 23.21	59.81 ± 8.83	66.10 ± 9.03
Beyond Graduate	2	59.37 ± 7.36	37.50 ± 4.41	56.25 ± 11.10	65.20 ± 12.06	79.79 ± 2.65
p-value	..	< 0.001	0.019	0.011	< 0.001	< 0.001
**Occupation**						
Student	30	70.55 ± 13.11	57.60 ±13.94	44.04 ± 26.72	54.59 ± 6.02	69.69 ±12.36
Employed	118	69.24 ± 12.57	59.90 ± 11.52	37.45 ± 22.65	54.30 ± 7.27	65.03 ± 10.25
Unemployed	412	71.12 ± 13.52	60.37 ± 10.13	25.98 ± 18.20	53.93 ± 7.27	61.27 ± 8.80
p-value	..	0.401	0.377	< 0.001	0.807	< 0.001

Variables	N (%)	SP	EKD	BKD	PC	MC
**Monthly Income**						
10,000	414	70.55 ± 13.38	60.12 ± 10.46	27.85 ± 19.23	53.50 ± 6.80	61.67 ± 9.40
25,000	122	71.46 ± 12.73	59.91 ± 10.89	30.85 ± 22.55	55.43 ± 7.89	64.05 ± 8.90
50,000	18	69.32 ± 16.50	61.63 ± 13.71	49.37 ± 24.69	57.46 ± 9.89	69.79 ± 14.48
1,00,000	6	69.09 ± 10.49	60.41 ± 12.60	43.75 ± 17.67	52.95 ± 5.42	67.29 ± 6.16
p-value	..	0.866	0.938	< 0.001	0.011	< 0.001
**Residential Status**						
Rural	263	71.68 ± 14.49	59.82 ± 10.91	24.02 ± 18.82	54.15 ± 7.41	61.15 ± 9.66
Urban	297	69.81 ± 12.10	60.39 ± 11.08	34.10 ± 20.86	53.95 ± 7.03	63.72 ± 9.40
p-value	..	0.096	0.529	< 0.001	0.748	0.002
**Diagnosis**						
Freshly Diagnosed	74	69.67 ± 10.99	57.43 ± 11.16	49.35 ± 24.19	52.49 ± 5.30	67.90 ± 11.78
Known Case	474	71.20 ± 13.37	60.71 ± 10.26	26.18 ± 17.84	54.27 ± 7.40	61.66 ± 8.65
Referred Case	12	56.77 ± 16.31	53.38 ± 17.65	31.87 ± 27.10	54.77 ± 8.92	63.12 ± 17.85
p-value	..	< 0.001	0.004	< 0.001	0.136	< 0.001
**History of Past Disease**						
Yes	507	70.64 ± 13.50	60.31 ± 10.52	27.13 ± 18.93	54.15 ± 7.39	61.90 ± 9.22
No	53	71.18 ± 11.33	58.31 ± 11.93	50.75 ± 23.08	53.05 ± 5.08	68.38 ± 11.18
p-value	..	0.778	0.193	< 0.001	0.293	< 0.001

SP - Symptoms, Problem list; **EKD - Effects of Kidney Disease**; **BKD - Burden of Kidney Disease**; **PC - Physical Component** (No. of items included: Work status (2), quality of social interaction (3), sexual function (2), social support (2), dialysis of staff encouragement (2)); **MC - Mental Component** (No. of items included: Cognitive function (3), sleep (4), patient satisfaction (1).
