# Supplementary material for: The impact of chronic kidney disease on health-related quality of life (HRQoL): key insights from a hospital-based cross-sectional study
Source: J Bras Nefrol. 2025 Jun 9;47(3):e20240229. doi: 10.1590/2175-8239-JBN-2024-0229en (PMC12176062; doi:10.1590/2175-8239-JBN-2024-0229en)
Supplement: Supplementary file 2 [file 2175-8239-jbn-47-3-e20240229-suppl2.pdf]

Braz. J. Nephrol.
https://doi.org/10.1590/2175-8239-JBN-2024-0229en

Supplementary Material to "The impact of chronic kidney disease on health-related quality of life (HRQoL): key insights from a hospital-based cross-sectional study"

Table S2 - ESRD Targeted Domains and Mean Summary Scores of Patients in Different Stages of CKD.

Scales	No. of Items	Cronbach's α	Stages of CKD					
			1 & 2 (n = 51)	3 (n = 70)	4 (n = 81)	5 (n = 358)	Total (n = 560)	*p*-value
SP	12	0.365	69.32 ± 12.78	75.20 ± 11.37	70.49 ± 14.82	70.05 ± 13.24	70.69 ± 13.30	0.023
EKD	8	0.847	61.58 ± 10.99	62.63 ± 9.50	60.49 ± 10.50	59.34 ± 10.81	60.12 ± 10.67	0.078
BKD	4	0.550	30.78 ± 18.64	26.69 ± 17.99	31.25 ± 22.44	29.27 ± 20.83	29.37 ± 20.54	0.550
PC	11	-0.025*	52.01 ± 6.00	54.16 ± 6.81	54.33 ± 7.50	54.24 ± 7.35	54.04 ± 7.20	0.215
MC	8	0.661	60.45 ± 9.48	63.04 ± 6.68	61.02 ± 10.58	63.04 ± 9.82	62.51 ± 9.60	0.133

***** The value is negative due to a negative average covariance among items. This violates reliability model assumptions.
