# Supplementary material for: The impact of chronic kidney disease on health-related quality of life (HRQoL): key insights from a hospital-based cross-sectional study
Source: J Bras Nefrol. 2025 Jun 9;47(3):e20240229. doi: 10.1590/2175-8239-JBN-2024-0229en (PMC12176062; doi:10.1590/2175-8239-JBN-2024-0229en)
Supplement: Supplementary file 3 [file 2175-8239-jbn-47-3-e20240229-suppl3.pdf]

Braz. J. Nephrol.
https://doi.org/10.1590/2175-8239-JBN-2024-0229en

Supplementary Material to "The impact of chronic kidney disease on health-related quality of life (HRQoL): key insights from a hospital-based cross-sectional study"

Table S3 - HRQoL Scores by Disease-Specific Characteristics, and Comparative Statistical Analysis of Mean Scores of SF-36 Items among patients with CKD according to Socio-Demographic Variables.

Variables	N (%)	PF	RP	BP	GH	EW	RE	SF	EF
**Patient Type**									
**OPD**	317	44.54 ± 18.29	18.22 ± 29.52	67.68 ± 20.41	37.83 ± 16.97	56.23 ± 9.03	23.76 ± 34.51	51.11 ± 15.98	42.44 ± 8.70
**IPD**	243	40.37 ± 18.60	16.98 ± 29.76	62.37 ± 18.56	35.45 ± 15.60	53.63 ± 8.97	19.89 ± 34.58	48.95 ± 13.23	41.36 ± 8.33
***p*-value**	..	0.008	0.623	0.002	0.089	< 0.001	0.189	0.089	0.136
**Age**									
**< 50**	228	47.48 ± 17.80	26.75 ± 34.99	67.52 ± 19.83	39.25 ± 16.34	56.88 ± 9.24	32.01 ± 39.86	51.83 ± 15.33	42.94 ± 8.45
**> 50**	332	39.47 ± 18.34	11.45 ± 23.33	63.90 ± 19.66	35.11 ± 16.28	53.88 ± 8.77	15.26 ± 28.52	49.03 ± 14.47	41.31 ± 8.56
***p*-value**	..	< 0.001	< 0.001	0.033	0.003	< 0.001	< 0.001	0.028	0.027
**Gender**									
**Male**	318	43.33 ±18.81	20.28 ± 31.41	66.26 ± 20.02	37.11 ± 16.98	55.28 ± 9.33	25.05 ± 36.94	49.60 ± 14.43	42.26 ± 8.99
**Female**	242	41.94 ± 18.61	14.26 ± 26.72	64.20 ± 19.46	36.40 ± 15.67	54.86 ± 8.75	18.18 ± 30.81	50.92 ± 15.45	41.59 ± 7.94
***p*-value**	..	0.379	0.017	0.222	0.613	0.585	0.020	0.300	0.357
**Marital Status**									
**Married**	517	41.77 ± 18.28	15.38 ± 26.87	65.04 ± 19.66	36.39 ± 16.20	54.77 ± 8.89	19.53 ± 32.06	50.04 ± 14.76	41.84 ± 8.51
**Unmarried**	43	54.30 ± 17.71	45.35 ± 44.07	69.35 ± 21.10	41.74 ± 18.35	59.07 ± 10.35	52.71 ± 47.27	51.67 ± 16.38	43.60 ± 8.95
***p*-value**	..	< 0.001	< 0.001	0.171	0.040	0.003	< 0.001	0.491	0.193
**Education**									
**Illiterate**	216	38.73 ± 19.28	9.61 ± 23.81	66.08 ± 20.53	32.93 ± 16.65	53.17 ± 8.85	11.57 ± 26.01	48.34 ± 15.87	39.77 ± 7.48
**Matric Pass**	267	45.75 ± 17.21	22.00 ± 31.49	66.09 ± 19.31	38.76 ± 16.25	56.01 ± 8.53	27.34 ± 36.63	51.07 ± 14.68	43.09 ± 8.80

Variables	N (%)	PF	RP	BP	GH	EW	RE	SF	EF
**Graduate**	75	42.93 ± 18.56	25.33 ± 33.00	60.17 ± 18.56	40.53 ± 13.93	57.12 ± 10.53	33.33 ± 41.00	52.25 ± 11.45	44.47 ± 9.17
**Beyond Graduate**	2	65.00 ± 28.28	25.00 ± 9.01	89.00 ± 15.55	52.50 ± 24.74	66.00 ± 8.48	33.33 ± 33.33	50.00 ± 35.35	37.50 ± 10.60
***p*-value**	..	< 0.001	< 0.001	0.034	< 0.001	< 0.001	< 0.001	0.126	< 0.001
**Occupation**									
**Student**	30	56.00 ± 18.21	57.50 ± 42.62	72.20 ± 21.40	42.67 ± 20.28	60.67 ± 10.71	65.55 ± 45.04	53.27 ± 17.52	43.50 ± 9.57
**Employed**	118	47.92 ± 19.78	23.94 ± 33.87	71.14 ± 20.91	41.57 ± 18.48	56.34 ± 10.14	37.00 ± 40.83	51.88 ± 14.89	42.92 ± 9.16
**Unemployed**	412	40.28±17.48	12.99 ± 24.25	63.22 ± 19.93	35.01 ± 15.10	54.34 ± 8.45	14.64 ± 27.38	49.45 ± 14.64	41.59 ± 8.28
***p*-value**	..	< 0.001	< 0.001	< 0.001	< 0.001	< 0.001	< 0.001	0.149	0.198
**Monthly Income**									
**10,000**	414	41.40 ± 18.13	14.73 ± 27.51	64.80 ± 19.46	35.43 ± 15.75	54.86 ± 8.81	17.06 ± 30.92	49.46 ± 15.00	41.45 ± 8.28
**25,000**	122	45.08 ± 18.35	24.18 ± 33.08	65.81 ± 20.82	39.43 ± 16.12	55.61 ± 8.85	32.24 ± 38.55	52.70 ± 13.27	43.28 ± 8.64
**50,000**	18	51.11 ± 22.46	37.50 ± 38.58	70.11 ± 19.97	47.78 ± 24.68	57.78 ± 14.53	55.55 ± 42.77	48.00 ± 21.03	43.89 ± 13.01
**1,00,000**	6	61.67 ± 19.66	29.17 ± 24.58	81.50 ± 15.25	45.00 ± 20.73	53.33 ± 12.30	61.11 ± 44.30	54.17 ± 12.71	45.83 ± 5.84
***p*-value**	..	0.003	< 0.001	0.144	< 0.001	0.483	< 0.001	0.151	0.089
**Residential Status**									
**Rural**	263	39.03 ± 18.29	14.54 ± 26.84	60.62 ± 19.06	34.92 ± 15.35	54.19 ± 9.27	15.71 ± 28.94	48.18 ± 14.45	40.36 ± 8.08
**Urban**	297	46.01 ± 18.14	20.45 ± 31.64	69.59 ± 19.50	38.47 ± 17.16	55.91 ± 8.84	27.72 ± 38.04	51.93 ± 15.05	43.40 ± 8.71
***p*-value**	..	< 0.001	0.018	< 0.001	0.011	0.026	< 0.001	0.003	< 0.001
**Diagnosis**									
**Freshly Diagnosed**	74	58.18 ± 18.84	46.62 ± 39.54	74.66 ± 19.45	46.28 ± 19.26	58.59 ± 11.60	53.60 ± 43.02	54.04 ± 16.96	44.39 ± 10.26
**Known Case**	474	40.62 ± 17.04	13.55 ± 25.28	63.92 ± 19.47	35.41 ± 15.18	54.58 ± 8.46	17.15 ± 30.28	50.00 ± 13.93	41.56 ± 8.17
**Referred Case**	12	30.83 ± 25.48	2.08 ± 7.21	65.50 ± 20.63	33.33 ± 24.89	54.00 ± 10.98	22.22 ± 35.76	33.17 ± 24.05	43.33 ± 9.61
***p*-value**	..	< 0.001	< 0.001	< 0.001	< 0.001	0.002	< 0.001	< 0.001	0.025
**History of Past Disease**									
**Yes**	507	40.82 ± 17.75	13.26 ± 25.10	64.69 ± 19.66	36.02 ± 15.81	54.75 ± 8.91	17.42 ± 30.60	49.50 ± 14.60	41.77 ± 8.34
**No**	53	61.04 ± 15.67	59.91 ± 35.80	71.91 ± 20.04	44.25 ± 20.05	58.42 ± 10.08	66.66 ± 38.67	56.53 ± 16.08	43.96 ± 10.20
***p*-value**	..	< 0.001	< 0.001	0.011	< 0.001	0.005	< 0.001	< 0.001	0.075
