# Supplementary material for: The impact of chronic kidney disease on health-related quality of life (HRQoL): key insights from a hospital-based cross-sectional study
Source: J Bras Nefrol. 2025 Jun 9;47(3):e20240229. doi: 10.1590/2175-8239-JBN-2024-0229en (PMC12176062; doi:10.1590/2175-8239-JBN-2024-0229en)
Supplement: Translation

# Translation: O impacto da doença renal crônica na qualidade de vida relacionada à saúde (QVRS): principais percepções de um estudo transversal de base hospitalar

**DOI:** 10.1590/2175-8239-JBN-2024-0229pt

**Authors:** Gautam Sahu, Pooja Arora, Pramil Tiwari, Sanjay D’Cruz, Anita Tahlan

**Affiliations:** 1 National Institute of Pharmaceutical Education and Research (NIPER), Department of Pharmacy Practice, Mohali, Punjab, Índia.; 2 National Institute of Pharmaceutical Education and Research (NIPER), Department of Pharmacoinformatics, Mohali, Punjab, Índia.; 3 Government Medical College and Hospital (GMCH), Department of General Medicine, Chandigarh, Índia.; 4 Government Medical College and Hospital (GMCH), Department of Pathology, Chandigarh, Índia.

**Keywords:** Insuficiência Renal Crônica, Qualidade de Vida, Fatores Sociodemográficos, Comorbidade.

## Abstract

**Introdução:**

Este estudo explora qualidade de vida relacionada à saúde (QVRS) em pacientes com DRC, analisando impacto de fatores sociodemográficos (idade, escolaridade, renda) e clínicos (estágio da doença, comorbidades, tipo de tratamento). O objetivo foi avaliar fatores contribuintes para melhora dos desfechos e para o bem-estar geral dos portadores de DRC.

**Métodos:**

Estudo transversal, realizado na clínica de nefrologia de um hospital público universitário, envolvendo 560 participantes selecionados aleatoriamente. O questionário KDQOL-SF^TM^ foi utilizado para coletar dados, analisados pelo SPSS (versão 20.0). Utilizou-se estatísticas descritivas para resumir características basais e o α de Cronbach para avaliar confiabilidade dos itens do questionário. As diferenças nos escores de QVRS entre grupos foram examinadas pelos testes t independentes e ANOVA, enquanto a análise de regressão foi usada para explorar associações entre variáveis.

**Resultados:**

O estudo analisou QVRS em 560 pacientes com DRC, com idade média de 53,32 anos e taxa de resposta de 81,27%. O escore médio de QVRS foi 32,03 ± 6,55. Homens predominaram nos estágios iniciais da DRC (72,5% nos estágios 1 e 2), enquanto mulheres predominaram no estágio 4. O índice de desemprego foi elevado (73,6%). Pacientes > 50 anos apresentaram escores mais altos *no domínio Burden of Kidney Disease* (BKD) (26,73 ± 19,36), enquanto pacientes mais jovens apresentaram melhores desfechos de saúde mental. Pacientes recém-diagnosticados apresentaram maiores escores nos domínios de QVRS do que casos já conhecidos. A escolaridade foi um preditor significativo de maior QVRS (p < 0,001), mas não a ocupação e a renda. Hipertensão (75,71%) e diabetes (39,64%) foram comorbidades comuns, ressaltando as influências socioeconômicas na QVRS na DRC.

**Conclusão:**

Este estudo destaca o forte impacto dos fatores sociodemográficos e clínicos, especialmente escolaridade, ocupação e estágio da doença, na QVRS dos pacientes com DRC. Intervenções precoces e manejo holístico da DRC, abordando questões clínicas e socioeconômicas, são fundamentais para melhorar o bem-estar do paciente.

## Introdução

No cenário contemporâneo dos cuidados com a saúde, a doença renal crônica (DRC) apresenta desafios significativos não apenas em termos de tratamento, mas também no bem-estar geral dos pacientes afetados^1,2^. Essa doença, caracterizada por um declínio progressivo da função renal, afeta milhões de pessoas em todo o mundo e impacta gravemente as dimensões física, mental e social da vida dos pacientes^3,4^. Os pacientes com DRC apresentam uma deterioração em sua QVRS, o que se destaca como uma preocupação essencial^5^. À medida que o cuidado com a saúde se volta para um atendimento mais centrado no paciente, a importância da avaliação e da melhoria da QVRS nesses indivíduos tem ganhado cada vez mais destaque nas comunidades clínicas e de pesquisa^6,7^.

Na esfera clínica, o estágio da DRC, a presença de comorbidades e o tipo de tratamento, seja por meio de diálise ou manejo conservador, também desempenham um papel crítico na determinação da QVRS^8,9^. Os pacientes em estágios avançados da doença, especialmente aqueles submetidos à diálise, geralmente relatam um declínio significativo em sua saúde física e mental. As exigências físicas da diálise, juntamente com as restrições por ela impostas à vida cotidiana, criam uma profunda sensação de sobrecarga^10,11^. Esses pacientes podem ainda enfrentar desafios cognitivos, distúrbios do sono e sentimentos de dependência, o que prejudica ainda mais a QVRS. Por outro lado, os pacientes em estágios iniciais da doença ou aqueles com manejo não dialítico têm maior probabilidade de manter uma QVRS melhor por mais tempo, embora ainda enfrentem a perspectiva iminente de progressão da doença^12,13^.

A avaliação da QVRS em pacientes com DRC é fundamental para fornecer uma compreensão holística de como a doença afeta os indivíduos para além dos parâmetros clínicos^14,15^. Ademais, as modalidades de tratamento, especialmente a diálise, introduzem desafios adicionais, como limitações de tempo, restrições alimentares e cargas emocionais, que afetam negativamente a QVRS^16,17^. Nesse contexto, compreender os fatores que influenciam a QVRS em pacientes com DRC torna-se essencial para adaptar intervenções que possam atenuar o ônus da doença e melhorar a QVRS geral dos pacientes^18,19^.

Os resultados disponíveis até o momento indicaram que diversos fatores sociodemográficos e clínicos influenciam a QVRS entre os pacientes com DRC. Idade, sexo, estado civil, nível de escolaridade, situação profissional, renda e local de residência são algumas das principais variáveis que desempenham um papel significativo na definição dos desfechos. Além disso, fatores específicos da doença, como o estágio da DRC, a duração da doença e o tipo de tratamento (diálise ou não diálise), influenciam ainda mais os escores de QVRS dos pacientes. A natureza multidimensional da QVRS ressalta a complexidade do manejo da DRC, pois requer uma abordagem integrada que vai além do mero manejo clínico, incluindo o apoio psicossocial^20–22^.

Dada a crescente importância da QVRS como um desfecho centrado no paciente, diversas ferramentas foram desenvolvidas para mensurá-la. Uma das mais amplamente utilizadas é o questionário *Kidney Disease Quality of Life-Short Form* (KDQOL-SF^TM^). O uso dessas ferramentas permite que os profissionais de saúde obtenham uma compreensão mais aprofundada de como a DRC afeta a vida dos pacientes e identifiquem áreas em que intervenções direcionadas possam fazer uma diferença significativa^23^.

Este estudo transversal teve como objetivo avaliar a QVRS entre pacientes com DRC e de que forma vários fatores sociodemográficos e clínicos influenciam essa variável. Ao compreender sistematicamente a relação entre esses fatores e a QVRS, o estudo visa avaliar fatores para melhorar os desfechos dos pacientes e aprimorar o bem-estar geral dos indivíduos que convivem com a DRC.

## Métodos

### Desenho do Estudo e População de Pacientes

Este estudo transversal recebeu aprovação do Comitê de Ética Institucional (IEC/78/2023-RI) e do Comitê de Ética Hospitalar (GMCH/IEC/2024/1137R).

O estudo foi realizado em um *Government Medical College and Hospital* (GMCH) em Chandigarh, Índia, de abril a outubro de 2024, com foco em pacientes com DRC. De um grupo de 689 pacientes que compareceram à clínica de nefrologia durante o período do estudo, 560 foram selecionados com base em critérios de inclusão e exclusão predefinidos. Os participantes elegíveis tinham entre 18 e 70 anos de idade e forneceram consentimento informado por escrito. Foram excluídos aqueles com distúrbios psiquiátricos, deficiências auditivas ou de fala, histórico de malignidade, perda de sangue recente ou ativa ou participação em outros ensaios clínicos de medicamentos, bem como gestantes e lactantes.

Dados detalhados foram coletados para cada participante, incluindo informações pessoais como nome da unidade, número UHID, especificações demográficas (por exemplo, nome, idade, sexo, endereço), bem como fatores clínicos e demográficos como pressão arterial, escolaridade, ocupação, estado civil, renda, condições de vida, hábitos sociais, estado de saúde e outras variáveis relevantes.

### Ferramenta e Procedimento de Coleta de Dados

Foi utilizado um instrumento autoaplicável estruturado e padronizado, incorporando o questionário *Kidney Disease Quality of Life* (KDQOL-SF™). Essa ferramenta consiste em duas partes: uma que aborda áreas específicas da doença renal em estágio terminal (DRET) e outra que compreende um questionário geral de saúde contendo 36 itens (SF-36). Embora a maioria dos participantes tenha preenchido o questionário de forma independente, os pesquisadores forneceram assistência para aqueles que precisaram de ajuda. A seção sobre DRET inclui diversas escalas, como 12 itens sobre sintomas e problemas (SP), 8 itens sobre os efeitos da doença renal (EKD), 4 itens que avaliam o ônus da doença renal (BKD), 11 itens que abrangem o componente físico (PC) e 8 itens focados no componente mental (MC). O componente SF-36 inclui 10 itens sobre funcionamento físico (PF), 4 sobre limitações de função devido à saúde física (RP), 2 sobre dor corporal (BP), 5 sobre estado geral de saúde (GH), 5 sobre bem-estar emocional (EW), 3 sobre papel emocional (RE), 2 sobre funcionamento social (SF) e 4 sobre energia e fadiga (EF). As respostas são convertidas em uma escala de 0 a 100, com pontuações mais elevadas indicando melhores desfechos de saúde. Alguns itens foram pré-codificados de tal forma que pontuações mais baixas significam uma condição de saúde mais favorável. Além disso, foram coletadas informações demográficas e clínicas sobre fatores como sexo, idade, escolaridade, ocupação, estado civil, renda mensal, local de residência e hábitos sociais^23^.

### Análise dos Dados

Os dados coletados pela pesquisa foram analisados utilizando a versão 20.0 do software *Statistical Package for the Social Sciences* (SPSS). Estatísticas descritivas, como média, desvios padrão, porcentagens e frequências, foram computadas para resumir as características sociodemográficas e relacionadas à saúde. A distribuição das respostas dos participantes do KDQOL-SF™ foi avaliada para identificar a proporção de pacientes que enfrentam dificuldades em cada dimensão. Os dados descritivos foram apresentados na forma de porcentagens e frequências. A consistência interna e a confiabilidade foram avaliadas usando o coeficiente *α* de Cronbach, calculado para cada subescala. Testes estatísticos, incluindo testes t para amostras independentes (para comparação das médias entre dois grupos) e ANOVA (para análise de três ou mais grupos), foram empregados para determinar diferenças significativas em dados demográficos, características relacionadas à saúde e QVRS. Além disso, foi realizada uma análise de regressão para verificar as associações entre as variáveis. Um nível de significância de 5% (*p* < 0,05) foi utilizado para determinar a significância estatística.

## Resultados

Os resultados do estudo revelaram variações significativas na QVRS entre pacientes com DRC com base em características sociodemográficas e clínicas. O impacto geral da doença renal na QVRS dos pacientes pode ser categorizado em quatro domínios principais: saúde física, saúde mental, funcionamento social e questões específicas relacionadas à doença renal (Figura 1). 

**Figura 1:** Quatro domínios-chave da saúde na doença renal.

A Tabela 1 apresenta uma visão abrangente das características sociodemográficas de pacientes com DRC nos diferentes estágios da doença. Uma maioria considerável (56,6%) foi tratada em regime ambulatorial (OPD, *outpatient department*), com os pacientes em estágio 5 representando a maior proporção desse grupo. Cinquenta e nove por cento dos pacientes tinham mais de 50 anos, principalmente nos estágios 3 e 5. Pacientes do sexo masculino foram mais comuns nos estágios iniciais (72,5% nos estágios 1 e 2) e a proporção de mulheres foi maior no estágio 4 da DRC.

**Tabela 1:** Análise comparativa dos escores de QVRS em variáveis sociodemográficas e específicas da doença em pacientes com DRC

Variáveis	Estágios da DRC				
	1 & 2 (n = 51)	3 (n = 70)	4 (n = 81)	5 (n = 358)	Total (n = 560)
Tipo de paciente					
OPD	31 (60,8%)	37 (52,9%)	37 (45,7%)	212 (59,2%)	317 (56,6%)
IPD	20 (39,2%)	33 (47,1%)	44 (54,3%)	146 (40,8%)	243 (43,3%)
Idade					
< 50	21 (41,2%)	24 (34,3%)	37 (45,7%)	146 (40,8%)	228 (40,7%)
> 50	30 (58,8%)	46 (65,7%)	44 (54,3%)	212 (59,2%)	332 (59,3%)
Sexo					
Masculino	37 (72,5%)	39 (55,7%)	35 (43,2%)	207 (57,8%)	318 (56,8%)
Feminino	14 (27,5%)	31 (44,3%)	46 (56,8%)	151 (42,2%)	242 (43,2%)
Estado Civil					
Casado	46 (90,2%)	64 (91,4%)	74 (91,4%)	333 (93,0%)	517 (92,3%)
Não Casado	5 (9,8%)	6 (8,6%)	7 (8,6%)	25 (7,0%)	43 (7,7%)
Escolaridade					
Analfabeto	18 (35,3%)	23 (32,9%)	30 (37,0%)	145 (40,5%)	216 (38,6%)
Ensino Médio Concluído	27 (52,9%)	38 (54,3%)	39 (48,1%)	163 (45,5%)	267 (47,7%)
Graduação	6 (11,8%)	9 (12,9%)	12 (14,8%)	48 (13,4%)	75 (13,4%)
Pós-graduação	0 (0,0)	0 (0,0)	0 (0,0)	2 (0,6%)	2 (0,4%)
Ocupação					
Estudante	4 (7,8%)	4 (5,7%)	4 (4,9%)	18 (5,0%)	30 (5,4%)
Empregado	8 (15,7%)	14 (20,0%)	17 (21,0%)	79 (22,1%)	118 (21,1%)
Desempregado	39 (76,5%)	52 (74,3%)	60 (74,1%)	261 (72,9%)	412 (73,6%)
Renda Mensal					
10.000	37 (72,5%)	56 (80,0%)	55 (67,9%)	266 (74,3%)	414 (73,9%)
25.000	9 (17,6%)	11 (15,7%)	24 (29,6%)	78 (21,8%)	122 (21,8%)
50.000	5 (9,8%)	2 (2,9%)	2 (2,5%)	9 (2,5%)	18 (3,2%)
1.00.000	0 (0,0)	1 (1,4%)	0 (0,0%)	5 (1,4%)	6 (1,1%)
Local de Residência					
Rural	19 (37,3%)	36 (51,4%)	39 (48,1%)	169 (47,2%)	263 (47,0%)
Urbana	32 (62,7%)	34 (48,6%)	42 (51,9%)	189 (52,8%)	297 (53,0%)
Diagnóstico					
Recém-diagnosticado	7 (13,7%)	7 (10,0%)	15 (18,5%)	45 (12,6%)	74 (13,2%)
Caso Conhecido	44 (86,3%)	62 (88,6%)	65 (80,2%)	303 (84,6%)	474 (84,6%)
Caso Encaminhado	0 (0,0%)	1 (1,4%)	1 (1,2%)	10 (2,8%)	12 (2,1%)
Histórico de Doenças Anteriores					
Sim	43 (84,3%)	69 (98,6%)	71 (87,7%)	324 (90,5%)	507 (90,5%)
Não	8 (15,7%)	1 (1,4%)	10 (12,3%)	34 (9,5%)	53 (9,5%)

A maioria dos pacientes em todos os estágios era casada (92,3%). Os níveis de escolaridade mostraram que 47,7% dos pacientes haviam concluído o ensino médio, embora o analfabetismo fosse mais prevalente nos estágios avançados, especialmente no estágio 5 (40,5%). O desemprego foi elevado em todos os estágios, com 73,6% dos pacientes desempregados. A maioria dos pacientes possuía renda mensal inferior a *₹*10.000 (73,9%). Os residentes urbanos superaram ligeiramente os rurais (53%), embora os pacientes de áreas rurais fossem mais prevalentes nos estágios 3 e 4. A maioria dos pacientes já eram casos conhecidos de DRC (84,6%) e uma porcentagem significativa (90,5%) tinha um histórico de doenças. Essas tendências foram particularmente notáveis nos estágios mais avançados da DRC.

### Prevalência de Hábitos Sociais em Pacientes com DRC

Os resultados mostram que o maior grupo em termos de hábitos sociais consistiu apenas de usuários de álcool, com 191 (34,10%) pessoas. O segundo maior grupo foi o de fumantes (tabagismo), com 166 (29,64%) pessoas. Os usuários de tabaco constituíram o menor grupo, com 108 (19,28%) indivíduos. A categoria “Outros”, que incluía diferentes hábitos, reuniu 157 (28,03%) pacientes. Esse padrão sugere que o consumo de álcool é o hábito social singular mais comum entre os pacientes com DRC, seguido pelo tabagismo.

A Figura 2 apresenta a prevalência de diferentes combinações de hábitos sociais entre os pacientes com DRC. O maior grupo foi composto por pacientes que eram fumantes e alcoólatras (T+A), com 76 (13,57%) indivíduos, seguido por aqueles que eram fumantes e se enquadravam na categoria “Outros” (T+O), com 69 (12,32%) pacientes. Por outro lado, o grupo com menor representação foi aquele com todos os quatro hábitos - tabagismo, consumo de álcool, uso de tabaco e outros hábitos (T+A+UT+O) - que incluiu apenas 17 pacientes (3,03%). A variabilidade no número de pacientes para cada grupo sugere que, embora o tabagismo e o consumo de álcool sejam os hábitos mais comuns entre os pacientes com DRC, a combinação de todos os quatro comportamentos é menos prevalente. Esses padrões podem ter implicações para a educação do paciente e para os programas de intervenção destinados a reduzir os riscos relacionados ao estilo de vida associados à DRC.

**Figura 2:** Distribuição dos hábitos sociais entre pacientes com DRC.

Abreviações – Tabagismo (T), Uso de Tabaco (UT), Alcoolismo (A), Outros (O). Notas – *Outros incluem – hábitos alimentares, sedentarismo, hidratação inadequada, uso de drogas e não adesão aos medicamentos.

### Principais Comorbidades da DRC

Os dados da Figura 3 mostram que a condição mais comum entre os pacientes com DRC foi a hipertensão (HT), afetando 424 (75,71%) pacientes. O diabetes foi a segunda condição mais comum, com 222 (39,64%) pessoas. Quarenta e dois (7,50%) pacientes apresentavam outras condições relacionadas aos rins, como doença renal policística (DRP) (7,50%), enquanto 47 (8,39%) tinham glomerulonefrite e 44 tinham pielonefrite (7,85%). Além disso, 190 (33,92%) pacientes foram classificados na categoria “Outras” condições e 28 (5,00%) pacientes declararam “Não sei” sobre sua condição subjacente. Essa distribuição sugere que a hipertensão e o diabetes são as comorbidades predominantes em pacientes com DRC, destacando a necessidade de estratégias de manejo e intervenção focadas nessas condições.

**Figura 3:** Causas da doença renal.

### Características Específicas da Doença: Ênfase na DRET

A análise dos escores de QVRS entre os pacientes com DRC revelou diferenças significativas com base nas características sociodemográficas e específicas da doença. Os pacientes ambulatoriais (OPD) demonstraram um manejo ligeiramente melhor dos sintomas (70,03 ± 13,43) e da saúde mental (63,10 ± 9,45) em comparação aos pacientes internados (IPD, *in-patients department*), que obtiveram escores marginalmente mais elevados no componente físico (53,57 ± 7,36). No entanto, os pacientes ambulatoriais (OPD) relataram um ônus da doença significativamente maior (BKD: 31,55 ± 21,13) em comparação com os pacientes internados (IPD: 26,52 ± 19,42) (*p* = 0,004). Os pacientes com mais de 50 anos apresentaram maior BKD (26,73 ± 9,36) em comparação com os pacientes mais jovens, que tiveram melhores escores no MC. Diferenças relacionadas ao sexo foram observadas apenas no PC, no qual os homens obtiveram escores mais elevados do que as mulheres (*p* = 0,004). Os pacientes não casados apresentaram um ônus da doença significativamente maior (40,31 ± 27,78), mas relataram melhor saúde mental em comparação aos pacientes casados (*p* < 0,001). Egressos relataram melhores desfechos de saúde física e mental (*p* < 0,001). Pacientes empregados obtiveram escores mais elevados em termos de saúde mental, mas também apresentaram maior BKD em comparação aos pacientes desempregados (*p* < 0,001). A renda também se mostrou um fator significativo, sendo que pacientes com maior renda relataram redução do BKD. Pacientes de áreas urbanas apresentaram maior BKD do que pacientes de áreas rurais, embora as diferenças nos escores de saúde física e mental não tenham sido significativas. Os pacientes com diagnóstico recente relataram maior BKD (*p* < 0,001), enquanto aqueles sem histórico prévio de doença apresentaram melhor MC. Esses achados ressaltam a importância da idade, estado civil, escolaridade, emprego, renda e status da doença na determinação da QVRS dos pacientes com DRC, sugerindo que intervenções personalizadas devem ser utilizadas para abordar essas vulnerabilidades específicas (Tabela S1).

### Domínios e Escores de DRET em Todos os Estágios da DRC

Foi realizada uma análise detalhada dos escores médios e da confiabilidade para vários domínios associados à DRET em diferentes estágios da DRC. A lista SP, que apresentou um escore de confiabilidade relativamente baixo (*α* de Cronbach = 0,365), demonstrou uma variação significativa entre os estágios da DRC, com os pacientes no estágio 3 relatando o escore médio mais elevado (75,20 ± 11,37) e os pacientes nos estágios 1 e 2, o mais baixo (69,32 ± 12,78), indicando uma diferença estatisticamente significativa (*p* = 0,023). Em contrapartida, o domínio EKD, com um alto índice de confiabilidade (*α* de Cronbach = 0,847), não apresentou diferenças significativas entre os estágios, com escores variando de 59,34 ± 10,81 no estágio 5 a 62,63 ± 9,50 no estágio 3 (*p* = 0,078). O domínio BKD apresentou confiabilidade moderada (*α* de Cronbach = 0,550) e não foram observadas diferenças significativas entre os estágios (*p* = 0,550). De forma semelhante, o PC apresentou baixa confiabilidade (*α* de Cronbach = 0,025), sem diferenças significativas entre os estágios (*p* = 0,215). O domínio MC, com confiabilidade moderada (*α* de Cronbach = 0,661), também não apresentou diferenças significativas entre os estágios (*p* = 0,133). Esses resultados destacam que, embora os sintomas variem significativamente entre os estágios da DRC, outros aspectos, como os efeitos e o ônus da doença, bem como os componentes físico e mental, permanecem relativamente estáveis ao longo da progressão da doença (Tabela S2).

### Comparação dos Escores de QVRS do SF-36 entre os Estágios da DRC

A Tabela 2 apresenta a análise de diversos domínios da QVRS em pacientes com DRC nos diferentes estágios, utilizando uma escala de 36 itens. O domínio PF, com alto índice de confiabilidade (*α* de Cronbach = 0,883), não apresentou diferenças significativas entre os estágios (p = 0,370), sendo os pacientes no estágio 3 os que relataram os escores mais elevados. Da mesma forma, o domínio RP, que mensura as limitações nas atividades diárias, apresentou alta confiabilidade (*α* de Cronbach = 0,782) e nenhuma diferença significativa entre os estágios (*p* = 0,628). O BP, com alta confiabilidade (*α* de Cronbach = 0,851), também se manteve estável entre os estágios (*p* = 0,889), embora os pacientes nos estágios 1 e 2 tenham relatado níveis de dor ligeiramente mais elevados. O domínio GH mostrou-se consistente em todos os estágios (*p* = 0,919; *α* de Cronbach = 0,576). O EW apresentou menor confiabilidade (*α* de Cronbach = 0,266), mas não demonstrou diferenças significativas (*p* = 0,254), embora os pacientes nos estágios 1 e 2 tenham relatado escores mais elevados. O domínio RE, que apresentou alta confiabilidade (*α* de Cronbach = 0,782), mostrou variabilidade significativa entre os estágios (*p* = 0,034), indicando que o funcionamento emocional é mais afetado pela progressão da DRC. Os domínios SF e a EF não apresentaram diferenças significativas entre os estágios, com escores de confiabilidade moderada e baixa, respectivamente. Esses achados sugerem que, embora a maioria das medidas de QVRS permaneça estável nos estágios da DRC, o funcionamento emocional é mais afetado à medida que a doença progride.

**Tabela 2:** Domínios SF-36 e escores médios resumidos de pacientes em diferentes estágios da DRC

Escalas	No. de Itens	*α* de Cronbach	Estágios da DRC					Valor de *p*
			1 & 2 (n = 51)	3 (n = 70)	4 (n = 81)	5 (n = 358)	Total (n = 560)	
PF	10	0,883	39,12 ± 20,53	45,00 ± 14,81	43,52 ± 21,14	42,63 ± 18,24	42,73 ± 18,53	0,370
RP	4	0,782	22,06 ± 34,14	15,36 ± 26,33	18,83 ± 29,45	17,25 ± 29,60	17,68 ± 29,60	0,628
BP	2	0,851	24,18 ± 37,16	19,52 ± 33,32	23,04 ± 34,01	22,06 ± 34,66	22,08 ± 34,56	0,889
GH	5	0,576	50,02 ± 16,11	50,67 ± 10,43	51,06 ± 15,88	49,89 ± 15,24	50,17 ± 14,88	0,919
EW	5	0,266	67,61 ± 21,47	63,56 ± 17,52	62,06 ± 21,80	66,16 ± 19,45	65,37 ± 19,73	0,254
RE	3	0,782	55,14 ± 8,47	55,26 ± 6,57	52,40 ± 9,43	55,68 ± 9,42	55,10 ± 9,08	0,034
SF	2	0,438	40,69 ± 8,18	43,14 ± 7,57	42,84 ± 8,94	41,73 ± 8,68	41,97 ± 8,55	0,311
EF	4	−0,238*	33,24 ± 18,10	38,64 ± 12,73	37,16 ± 16,16	36,87 ± 16,84	36,80 ± 16,41	0,344

Abreviações – PF: Funcionamento Físico, RP: Papel Físico, BP: Dor Corporal, GH: Estado Geral de Saúde, EW: Bem-estar Emocional, RE: Papel Emocional, SF: Funcionamento Social, EF: Energia/Fadiga.

Notas – *O valor é negativo devido a uma covariância média negativa entre os itens. Isso viola as premissas do modelo de confiabilidade.

### Impacto das Características dos Pacientes nos Escores de QVRS

Foi realizada uma análise comparativa dos escores de QVRS em diversos domínios em pacientes com DRC, com base em variáveis sociodemográficas e específicas da doença. O OPD apresentou escores significativamente mais elevados em funcionamento físico (*p* = 0,008) e dor corporal (*p* = 0,002) em comparação com o IPD. O EW (*p* < 0,001) também foi maior em pacientes OPD, enquanto outros domínios não apresentaram diferença significativa. Pacientes com idade < 50 anos apresentaram escores significativamente mais elevados em vários domínios, incluindo PF (*p* < 0,001), RP (*p* < 0,001), GH (*p* = 0,003) e EW (*p* < 0,001), entre outros, em comparação com pacientes > 50 anos. Embora não tenham sido observadas diferenças significativas entre homens e mulheres na maioria dos domínios, RP (*p* = 0,017) e RE (*p* = 0,020) foram significativamente mais elevados nos homens. Pacientes não casados apresentaram escores significativamente mais elevados em PF (*p* < 0,001), RP (*p* < 0,001), GH (*p* = 0,040) e EW (*p* = 0,003) em comparação aos pacientes casados. Um nível de escolaridade mais elevado foi associado a escores significativamente melhores em quase todos os domínios, incluindo PF (*p* < 0,001), RP (*p* < 0,001) e GH (*p* < 0,001). Pacientes empregados e estudantes relataram escores significativamente melhores na maioria dos domínios em comparação com pacientes desempregados, com PF (*p* < 0,001), RP (*p* < 0,001) e BP (*p* < 0,001) apresentando as maiores diferenças. Renda mais elevada esteve associada a desfechos significativamente melhores em PF (*p* = 0,003), RP (*p* < 0,001) e GH (*p* < 0,001). Pacientes residentes em áreas urbanas apresentaram escores mais elevados em PF (*p* < 0,001), BP (*p* < 0,001) e EW (*p* = 0,026), enquanto pacientes de áreas rurais apresentaram escores menores em todos esses domínios. Pacientes recém-diagnosticados apresentaram escores significa­tivamente mais elevados em todos os domínios em comparação com casos já conhecidos, com PF (*p* < 0,001) e RP (*p* < 0,001) exibindo as maiores diferenças. Pacientes sem histórico de doença anterior apresentaram escores significativamente melhores em PF (*p* < 0,001), RP (*p* < 0,001) e EW (*p* = 0,005). Em conclusão, a análise revela que fatores sociodemográficos como idade, estado civil, escolaridade e renda, juntamente com características específicas da doença, influenciam significativamente a QVRS em pacientes com DRC. Pacientes mais jovens, não casados, com escolaridade e emprego, bem como aqueles sem histórico prévio de doenças, tendem a relatar melhores desfechos de saúde em múltiplos domínios (Tabela S3).

### Comportamentos de Risco por Perfil Demográfico

A Tabela 3 apresenta uma análise comparativa dos padrões de comportamento de risco, como tabagismo, consumo de álcool, uso de tabaco e outros comportamentos não saudáveis entre pacientes com DRC, categorizados por idade e sexo. Entre os homens, aqueles com mais de 50 anos apresentaram maior prevalência de tabagismo (39,2%) em comparação aos homens mais jovens, com menos de 50 anos (31,1%). No entanto, o consumo de álcool foi mais comum em homens mais jovens (52,3%) do que em homens mais velhos (37,6%). O uso de tabaco, por outro lado, foi significativamente maior em homens com mais de 50 anos (24,7%) em comparação aos mais jovens (6,1%). Outros comportamentos não saudáveis, como maus hábitos alimentares, sedentarismo e não adesão à medicação, foram ligeiramente mais prevalentes em homens mais velhos (30,1%) do que entre os mais jovens (28,8%). Entre as mulheres, o tabagismo e o consumo de álcool foram consideravelmente inferiores aos observados nos homens. Mulheres mais jovens (< 50 anos) apresentaram menor prevalência de tabagismo (9,4%) e consumo de álcool (15,6%) do que seus pares masculinos. De modo semelhante, mulheres mais velhas (> 50 anos) apresentaram baixas taxas de tabagismo (9,6%) e consumo de álcool (10,3%), com apenas pequenas diferenças em relação às mulheres mais jovens. O uso de tabaco foi ligeiramente maior entre as mulheres mais velhas (8,2%) em comparação às mulheres mais jovens (7,3%). Outros comportamentos não saudáveis foram mais comuns em mulheres mais velhas (26,0%) do que entre as mais jovens (21,9%). Em conclusão, os homens apresentaram maior prevalência de tabagismo, consumo de álcool e uso de tabaco quando comparados às mulheres em todas as faixas etárias. Além disso, os comportamentos de risco tenderam a aumentar com a idade em ambos os sexos. Esses achados destacam a importância de intervenções direcionadas para reduzir comportamentos de risco, particularmente entre pacientes do sexo masculino, mais velhos, com DRC, que apresentaram taxas mais elevadas de uso de tabaco e álcool.

**Tabela 3:** Padrões de comportamento de risco por idade e sexo

Variáveis	Idade (anos)	Fumante N (%)	Usuário de álcool N (%)	Usuário de tabaco N (%)	Outros N (%)
Sexo					
Masculino	< 50	41 (31,1)	69 (52,3)	8 (6,1)	38 (28,8)
	> 50	73 (39,2)	70 (37,6)	46 (24,7)	56 (30,1)
Valor de *p*		0,134	0,010	0,001	0,799
Feminino	< 50	9 (9,4)	15 (15,6)	7 (7,3)	21 (21,9)
	> 50	14 (9,6)	15 (10,3)	12 (8,2)	38 (26,0)
Valor de *p*		0,956	0,217	0,793	0,462

Nota – Outros incluem: maus hábitos alimentares, sedentarismo, hidratação inadequada, uso de drogas e não adesão a medicamentos.

### Padrões de Comorbidade por Idade e Sexo

A Tabela 4 apresenta uma análise detalhada da prevalência de comorbidades, como HT, diabetes, doença renal policística (DRP), glomerulonefrite, pielonefrite e outras condições relacionadas, categorizadas por idade e sexo entre pacientes com DRC. Entre os homens, a hipertensão foi mais comum naqueles com idade > 50 anos (79,6%) em comparação aos com idade < 50 anos (72,0%). O diabetes apresentou um aumento estatisticamente significativo na prevalência entre homens mais velhos (45,7%) em comparação aos mais jovens (32,6%) (*p* = 0,019). A DRP e a glomerulonefrite foram ligeiramente mais frequentes em homens mais jovens, embora essas diferenças não fossem estatisticamente significativas. A pielonefrite foi significativamente mais prevalente entre os homens mais jovens (15,2%) em comparação àqueles com mais de 50 anos (5,9%) (*p* = 0,006). Para as mulheres, a prevalência de hipertensão foi maior naquelas com mais de 50 anos (80,1%) em comparação às mulheres mais jovens (66,7%) (*p* = 0,018). O diabetes foi significativamente mais prevalente em mulheres mais velhas (47,3%) do que naquelas com menos de 50 anos (26,0%) (*p* < 0,001). A DRP e a glomerulonefrite foram mais comuns em mulheres mais jovens, sendo que a DRP apresentou uma taxa significativamente mais elevada entre mulheres mais jovens (14,6%) em comparação com mulheres mais velhas (4,1%) (*p* = 0,004). A pielonefrite foi ligeiramente mais prevalente entre mulheres mais jovens (8,3%) do que entre mulheres mais velhas (3,4%), mas a diferença não foi estatisticamente significativa. Em conclusão, a análise revelou que a hipertensão e o diabetes foram mais prevalentes entre pacientes mais velhos com DRC, particularmente entre mulheres. Homens e mulheres mais jovens tendem a apresentar maior prevalência de pielonefrite e DRP. Esses achados sugerem que a distribuição das comorbidades varia simultaneamente com a idade e o sexo, destacando a necessidade de intervenções médicas personalizadas para abordar os riscos específicos de comorbidades em diferentes grupos demográficos.

**Tabela 4:** Análise comparativa de comorbidades

Variáveis	Idade (anos)	HT N (%)	Diabetes N (%)	DRP N (%)	Glomerulonefrite N (%)	Pielonefrite N (%)	Outros N (%)	“Não sei” N (%)
Sexo								
Masculino	< 50	95 (72,0)	43 (32,6)	12 (9,1)	15 (11,4)	20 (15,2)	40 (30,3)	7 (5,3)
	> 50	148 (79,6)	85 (45,7)	10 (5,4)	12 (6,5)	11 (5,9)	56 (30,1)	9 (4,8)
Valor de *p*		0,116	0,019	0,198	0,112	0,006	0,970	0,852
Feminino	< 50	64 (66,7)	25 (26,0)	14 (14,6)	8 (8,3)	8 (8,3)	39 (40,6)	2 (2,1)
	> 50	117 (80,1)	69 (47,3)	6 (4,1)	12 (8,2)	5 (3,4)	55 (37,7)	10 (6,8)
Valor de *p*		0,018	0,001	0,004	0,975	0,098	0,645	0,095

Nota – Outros incluem: ITU, pancreatite, uso de drogas, hepatite, edema pedal e obstrução.

### Relevância dos Fatores que Influenciam a QVRS

A análise de regressão apresentada na Tabela 5 mostra a associação entre a QVRS e diversos fatores. O modelo explica cerca de 14,1% da variação na QVRS, com o modelo geral sendo estatisticamente significativo (*F* = 8,147, *p* < 0,001). Entre as variáveis, o tipo de paciente apresentou um impacto negativo significativo na QVRS (*B* = −8,79, *p* = 0,002), sugerindo que pacientes em diferentes categorias (provavelmente pacientes internados) apresentam escores de QVRS mais baixos em comparação aos demais. A idade, embora apresentando um coeficiente positivo (*B* = 2,82), não influenciou significativamente a QVRS (*p* = 0,394), assim como o sexo (*B* = −0,96, *p* = 0,738). Da mesma forma, o estado civil, embora tenha um efeito positivo (*B* = 3,91), não foi estatisticamente significativo (*p* = 0,613), indicando que teve pouco impacto na QVRS. A escolaridade, no entanto, surge como um preditor positivo significativo (*B* = 10,71, *p* < 0,001), demonstrando que níveis mais elevados de escolaridade estão fortemente associados a uma melhor QVRS. Em contrapartida, a ocupação e a renda mensal, embora associadas positivamente à QVRS (*B* = 1,62 e *B* = 2,97, respectivamente), não foram estatisticamente significativas (*p* = 0,683 e *p* = 0,349), sugerindo que a situação profissional e os níveis de renda não afetaram significativamente a QVRS nesta amostra. O local de residência também não apresentou efeito significativo (*B* = 3,66, *p* = 0,244). O tipo de diagnóstico desempenhou um papel crucial na determinação da QVRS, com um impacto negativo significativo (*B* = -13,36, *p* = 0,007), indicando que determinados diagnósticos estão associados a uma pior QVRS. Curiosamente, um histórico de doença anterior influenciou positivamente a QVRS (*B* = 13,49, *p* = 0,04), possivelmente porque pacientes com histórico de doenças podem desenvolver melhores mecanismos de enfrentamento ou estratégias de manejo. O estágio da doença, no entanto, não apresentou efeito significativo na QVRS (*B* = 0,020, *p* = 0,987), sugerindo que a progressão da doença por si só não altera substancialmente a QVRS neste modelo. De modo geral, a escolaridade e o diagnóstico emergiram como os fatores mais significativos que afetam a QVRS, enquanto outras variáveis, como idade, sexo, ocupação e renda, apresentam influência mínima.

**Tabela 5:** Análise de regressão mostrando a associação entre QVRS e diversas variáveis demográficas e clínicas

Variáveis	*B*	Erro padrão	*t*	Significância
Tipo de Paciente	−8,79	2,84	−3,09	0,002
Idade	2,82	3,31	0,85	0,394
Sexo	−0,96	2,89	−0,33	0,738
Estado Civil	3,91	7,73	0,50	0,613
Escolaridade	10,71	2,58	4,13	0,001
Ocupação	1,62	3,96	0,40	0,683
Renda Mensal	2,97	3,17	0,93	0,349
Local de Residência	3,66	3,14	1,16	0,244
Diagnóstico	−13,36	4,90	−2,72	0,007
Histórico de Doenças Anteriores	13,49	6,55	2,05	0,040
Estágios	0,020	1,23	0,017	0,987

Notas – *R* ^2^ = 0,141, *F* = 8,147, e *p* < 0,001.

## Discussão

Os resultados deste estudo revelam importantes *insights* sobre a QVRS de pacientes com DRC, destacando o impacto profundo de fatores socio­demográficos e clínicos nos desfechos dos pacientes. Os achados ressaltam a natureza multifacetada do manejo da DRC, em que não apenas indicadores clínicos, mas também dimensões sociais e psicológicas devem ser considerados para melhorar de forma abrangente o bem-estar dos pacientes.

Um achado significativo deste estudo foi a variação nos escores de QVRS entre os diferentes estágios da DRC, com pacientes em estágios avançados, particularmente aqueles em diálise, apresentando QVRS significativamente mais baixa em comparação com aqueles em estágios iniciais^4,24^. Esse declínio, especialmente no bem-estar físico e emocional, é amplamente atribuído ao impacto cumulativo dos sintomas relacionados à DRC, como fadiga, dor e comprometimentos cognitivos^25^. Além disso, a natureza exigente da diálise, incluindo tratamentos frequentes, restrições alimentares e redução da atividade física, agrava ainda mais a deterioração da qualidade de vida. Neste estudo, os pacientes internados apresentaram escores mais baixos nos domínios BKD e PC, o que está em consonância com pesquisas anteriores que associam estágios avançados de DRC a uma redução significativa da QVRS^25,26,27,28^.

Além disso, fatores sociodemográficos desempen­haram um papel fundamental na determinação dos desfechos da QVRS. A idade emergiu como um determinante significativo, com pacientes mais jovens (< 50 anos) relatando melhor funcionamento físico e bem-estar emocional em comparação aos pacientes mais velhos (> 50 anos)^19,29^. Isso pode ser atribuído à maior resiliência física e adaptabilidade psicológica dos pacientes mais jovens, enquanto pacientes mais velhos podem enfrentar mais comorbidades e limitações físicas que comprometem sua QVRS^30,31^. Curiosamente, pacientes não casados apresentaram escores mais elevados em diversos domínios, incluindo bem-estar emocional e funcionamento físico, em comparação aos seus homólogos casados. Isso pode ser atribuído a diferenças nos sistemas de apoio social ou às responsabilidades e ao estresse adicionais que acompanham a vida familiar, especialmente para aqueles que lidam com uma doença crônica^32^.

O nível de escolaridade e a situação profissional também influenciaram significativamente os escores de QVRS. Pacientes com níveis mais elevados de escolaridade e aqueles que estavam empregados relataram melhor QVRS geral^33,34^. Esses grupos provavelmente se beneficiam de um melhor acesso a recursos de saúde, melhor alfabetização em saúde e um senso de propósito ou rotina proporcionado pelo emprego^35^. Em contrapartida, pacientes desempregados e aqueles com níveis de escolaridade mais baixos apresentaram maiores chances de enfrentar restrições financeiras, o que pode limitar o acesso aos cuidados de saúde e aumentar o estresse psicológico, diminuindo ainda mais sua QVRS^36^. Além disso, a renda influenciou os escores de QVRS, com pacientes de maior renda apresentando melhores desfechos em domínios como funcionamento físico e dor corporal. Esse achado destaca as desigualdades socioeconômicas que persistem no acesso aos cuidados de saúde e nos desfechos entre pacientes com DRC^35,37^.

A influência de fatores clínicos, como a duração da doença e a presença de comorbidades, tais como hipertensão e diabetes, esteve fortemente associada à redução da QVRS, especialmente em pacientes mais velhos^19,38^. O ônus cumulativo do manejo de múltiplas condições crônicas contribui para piores desfechos de saúde física e geral. No entanto, pacientes recém-diagnosticados relataram melhor QVRS, provavelmente devido à menor quantidade de sintomas e menos desgaste psicológico nos estágios iniciais da DRC^24^. Isso destaca a necessidade de intervenção precoce e apoio contínuo para manter a QVRS à medida que a doença avança^39^.

Em resumo, o estudo demonstra que o manejo da DRC requer uma abordagem personalizada que considere o estágio da doença, fatores sociodemográficos e comorbidades. Intervenções personalizadas, como maior apoio psicossocial para pacientes mais jovens e cuidados integrados para pacientes mais velhos com múltiplas condições, são essenciais. Abordar as barreiras socioeconômicas também é crucial para reduzir as disparidades na QVRS. Em última análise, melhorar a QVRS em pacientes com DRC requer uma abordagem holística e centrada no paciente, que vá além dos cuidados clínicos, para enfrentar desafios psicossociais e econômicos mais amplos.

### Limitações

A natureza de centro único deste estudo pode limitar a generalização dos achados. A falta de acompanhamento longitudinal restringe a compreensão das mudanças na QVRS ao longo do tempo e a ausência de dados sobre o impacto do suporte emocional e psicológico pode afetar a validade dos resultados.

### Pontos Fortes

Um dos principais pontos fortes deste estudo reside na grande dimensão da amostra, o que aumenta a confiabilidade e a robustez dos achados. O foco do estudo em variáveis sociodemográficas e clínicas proporciona uma compreensão abrangente de como diversos fatores influenciam a QVRS, oferecendo *insights* para intervenções direcionadas. Além disso, o desenho transversal destaca as diferentes necessidades entre os estágios da DRC.

### Direções para Pesquisas Futuras

Trabalhos futuros devem considerar uma abordagem longitudinal para o acompanhamento da QVRS ao longo do tempo, desenhos multicêntricos para uma aplicabilidade mais ampla e a análise do papel das intervenções de saúde mental e do suporte social na melhoria da QVRS. A integração de percepções qualitativas aprimoraria ainda mais as intervenções centradas no paciente.

## Conclusões

Este estudo destaca o impacto significativo dos fatores sociodemográficos e clínicos na QVRS em pacientes com DRC, analisando determinantes-chave, como idade, escolaridade, situação profissional, nível de renda e comorbidades. O estudo proporciona uma compreensão mais aprofundada dos desafios mais amplos enfrentados pelos pacientes com DRC e identifica áreas potenciais para intervenções direcionadas.

Os achados evidenciam o declínio substancial da QVRS à medida que a doença progride, particularmente nos estágios avançados que requerem diálise. Pacientes em estágios mais avançados apresentam deterioração notável no bem-estar físico, emocional e social, enfatizando a necessidade de uma abordagem abrangente para o manejo da DRC.

Os fatores sociodemográficos desempenham um papel fundamental na determinação dos desfechos da QVRS, com pacientes mais jovens, com maior nível de escolaridade e empregados relatando melhor QVRS, enquanto indivíduos mais velhos, desempregados e com menor renda enfrentam maiores dificuldades.

Além disso, a presença de comorbidades, como hipertensão e diabetes, agrava ainda mais o declínio da QVRS, principalmente entre pacientes mais velhos. Adicionalmente, pacientes recém-diagnosticados apresentaram melhor QVRS em comparação àqueles com histórico mais longo da doença, ressaltando a importância da detecção precoce e da intervenção oportuna.

Este estudo conclui que o manejo da DRC deve ir além das intervenções clínicas para englobar fatores psicossociais, econômicos e emocionais. Uma abordagem abrangente e centrada no paciente, que integre iniciativas educacionais, suporte psicossocial e melhor acesso aos cuidados de saúde, é essencial para melhorar a QVRS. Em última análise, uma estrutura multidimensional que combine cuidados médicos, psicológicos e sociais é crucial para atender às necessidades complexas dos pacientes com DRC e melhorar seu bem-estar geral.

## Data Availability

Este estudo envolveu a análise de conjuntos de dados de acesso público. Os conjuntos de dados utilizados e/ou analisados na presente pesquisa podem ser obtidos junto ao autor correspondente mediante solicitação razoável.

## Material Suplementar

O seguinte material online está disponível para este artigo:

Tabela S1 – Analysis of HRQoL Scores Based on Disease-Specific Characteristics and Comparative Statistical Evaluation of Mean Scores in ESRD-Focused Areas Among CKD Patients, Stratified by Socio-Demographic Variables.

Tabela S2 – ESRD Targeted Domains and Mean Summary Scores of Patients in Different Stages of CKD.

Tabela S3 – HRQoL Scores by Disease-Specific Characteristics and Comparative Statistical Analysis of Mean Scores of SF-36 Items among Patients with CKD according to the Socio-Demographic Variables.
